# Neuromedin U receptor 1 deletion leads to impaired immunotherapy response and high malignancy in colorectal cancer

**DOI:** 10.1016/j.isci.2024.110318

**Published:** 2024-06-20

**Authors:** Yulai Zhou, Xiangyang Zhang, Yan Gao, Yinghui Peng, Ping Liu, Yihong Chen, Cao Guo, Gongping Deng, Yanhong Ouyang, Yan Zhang, Ying Han, Changjing Cai, Hong Shen, Le Gao, Shan Zeng

**Affiliations:** 1 Department of Oncology, Xiangya Hospital, Central South University, Changsha, Hunan 410008, China; 2 Department of Microbiology, Immunology & Molecular Genetics, University of Texas Long School of Medicine, UT Health Science Center, San Antonio, TX 78229, USA; 3 Department of Hematology, The First Affiliated Hospital of Zhengzhou University, Zhengzhou, Henan 450052, China; 4 National Clinical Research Center for Geriatric Disorders, Xiangya Hospital, Central South University, Changsha, Hunan 410008, China; 5 Department of Emergency, Hainan General Hospital, Hainan Affiliated Hospital of Hainan Medical University, Haikou, Hainan 570311, China; 6 Department of Oncology, Yueyang People’s Hospital, Yueyang Hospital Affiliated to Hunan Normal University, Yueyang, Hunan 414000, China

**Keywords:** Bioinformatics, Cancer, Microenvironment, Molecular biology, Therapeutics

## Abstract

Colorectal cancer (CRC) exhibits significant heterogeneity, impacting immunotherapy efficacy, particularly in immune desert subtypes. Neuromedin U receptor 1 (NMUR1) has been reported to perform a vital function in immunity and inflammation. Through comprehensive multi-omics analyses, we have systematically characterized NMUR1 across various tumors, assessing expression patterns, genetic alterations, prognostic significance, immune infiltration, and pathway associations at both the bulk sequencing and single-cell scales. Our findings demonstrate a positive correlation between NMUR1 and CD8^+^ T cell infiltration, with elevated NMUR1 levels in CD8^+^ T cells linked to improved immunotherapy outcomes in patients with CRC. Further, we have validated the NMUR1 expression signature in CRC cell lines and patient-derived tissues, revealing its interaction with key immune checkpoints, including lymphocyte activation gene 3 and cytotoxic T-lymphocyte-associated protein 4. Additionally, NMUR1 suppression enhances CRC cell proliferation and invasiveness. Our integrated analyses and experiments open new avenues for personalized immunotherapy strategies in CRC treatment.

## Highlights

• NMUR1 in CD8^+^ T cells correlates positively with immunotherapy outcomes in CRC
• NMUR1 deletion increases colorectal cancer cell proliferation and invasion *in vitro*
• NMUR1 is a biomarker for both immunotherapy response and prognosis in CRC

## Graphical abstract

**Figure:**
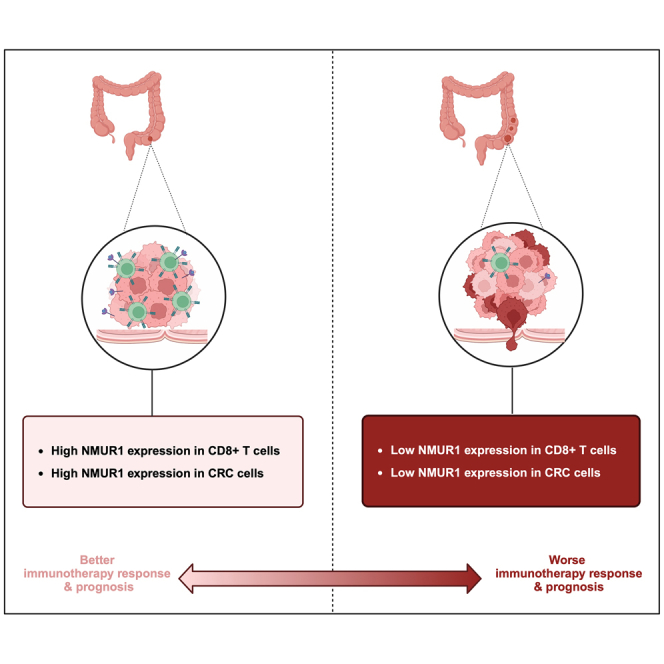

## Introduction

Neuromedin U (NMU), a highly conserved neuropeptide, is known to regulate smooth muscle contraction, intestinal motility, and blood flow.^1^^,^^2^ NMU receptors (NMURs), belonging to the G-protein-coupled receptor (GPCR) family, exhibit distinct expression patterns^3^^,^^4^; NMUR1 is primarily peripheral, while NMUR2 is central nervous system-oriented.^5^^,^^6^ Besides physiological function, accumulated studies have identified that NMU has a significant impact on tumorigenesis and metastasis in colorectal cancer (CRC), lung cancer, renal cancer, breast cancer, hepatocellular cancer, endometrial cancer, and myeloid leukemia.^7^^,^^8^^,^^9^^,^^10^^,^^11^^,^^12^^,^^13^ And NMUR1 is expressed on various cell types, including macrophages and endothelial cells, known to be active modulators of the tumor microenvironment.^6^ The interaction between NMU and Group 2 innate lymphoid cells (ILC2s), which are implicated in cytokine secretion and express NMUR1, underscores the importance of NMU-NMUR1 signaling in immunity and inflammation.^14^^,^^15^^,^^16^^,^^17^^,^^18^ Furthermore, through an NMUR1-dependent manner, NMU was found to regulate anti-tumor activity of CD8^+^ T cells, which are the most powerful effectors in the anticancer immune response and form the backbone of current successful cancer immunotherapies.^19^^,^^20^ NMU has also been shown to be a candidate biomarker and therapeutic target in drug resistance.^21^^,^^22^ Although NMUR1 is widely expressed on many immune cells, such as macrophages, endothelial cells, ILC2, and CD8^+^ T cells, especially in the gastrointestinal tract, and is known to interact with NMU in the tumor microenvironment and drug resistance, its role in the CRC microenvironment and treatment remains unclear.

Tumor microenvironment (TME) is a dynamic and complicated component consisting of stromal cells, immune cells, and signaling molecules, except for tumor cells.^23^^,^^24^ Accumulating researches suggest that the ratio of tumor-infiltrating immune cells, stromal component, and cytokines have a significant impact on the regulation of tumor progression, evasion, metastasis, and evolution.^24^^,^^25^^,^^26^^,^^27^^,^^28^^,^^29^^,^^30^ The complicated correlation between tumor and immune system influences the efficiency of traditional anticancer therapy strategies, such as surgery, radiotherapy, and chemotherapy. This interplay accelerates advances in tumor therapeutic methods that function by overcoming tumor evasion and optimizing the clinical benefit.^31^^,^^32^^,^^33^^,^^34^^,^^35^ Immunotherapy is a novel anticancer therapy that suppresses cancer development by the activation of the immune system.^36^^,^^37^ As a promising therapeutic technique in immunotherapy, immune checkpoint inhibitors (ICIs) have been acknowledged to be involved in conquering tumor microenvironment immunosuppression and enhancing the immunological anti-cancer responses by blocking inhibitory immune checkpoints,^38^^,^^39^^,^^40^^,^^41^ such as cytotoxic T lymphocyte antigen-4 (CTLA4)^42^ and programmed cell death protein 1 (PD-1),^43^ which have shown survival benefits in clinical trials. Among emerging treatment strategies for patients with cancer, few have progressed as rapidly as neoadjuvant immune checkpoint blockade (ICB). Combining neoadjuvant chemotherapy with PD-1 blockade has shown increased expression of CD8^+^ T cell activation markers and reduced expression of markers related to immunosuppression.^44^ Neoadjuvant ICB can induce a diverse range of inflammatory infiltrates, including CD8^+^ tumor-infiltrating lymphocytes (TIL), neutrophils, and plasma cells. It also has the potential to upregulate certain immune checkpoint pathways and remodel cancer-associated fibroblasts in the tumor bed.^45^^,^^46^ Despite these promising developments, there’s a pressing need to identify new biomarkers that can stratify patients based on their sensitivity to ICB and predict therapy response.^47^

Based on the high expression of NMUR1 in the gastrointestinal tract^1^ and its close association with immune cells,^17^ we hypothesized that NMUR1 played a crucial role in CRC. To identify the potential of NMUR1 as a biomarker in CRC, our study first conducted a comprehensive analysis of the function of NMUR1 in pan-cancer, including NMUR1 expression profiles, epigenetic alteration, the impact of survival, correlation with immune infiltration and associated pathways in 33 types of cancer. Given the important role of NMUR1 in pan-cancer, we aimed to investigate immune signatures and the function of NMUR1 in CRC cell lines and patient tissues by performing single-cell RNA sequencing and functional experiments. And we also revealed the prognosis value and immune signature of NMUR1 in the CRC patient cohort. In summary, this study uncovered that NMUR1 is a promising biomarker for immunotherapy response and function as a suppressor in CRC.

## Results

### Neuromedin U receptor 1 expression in human pan-cancer

**Figure 1:**
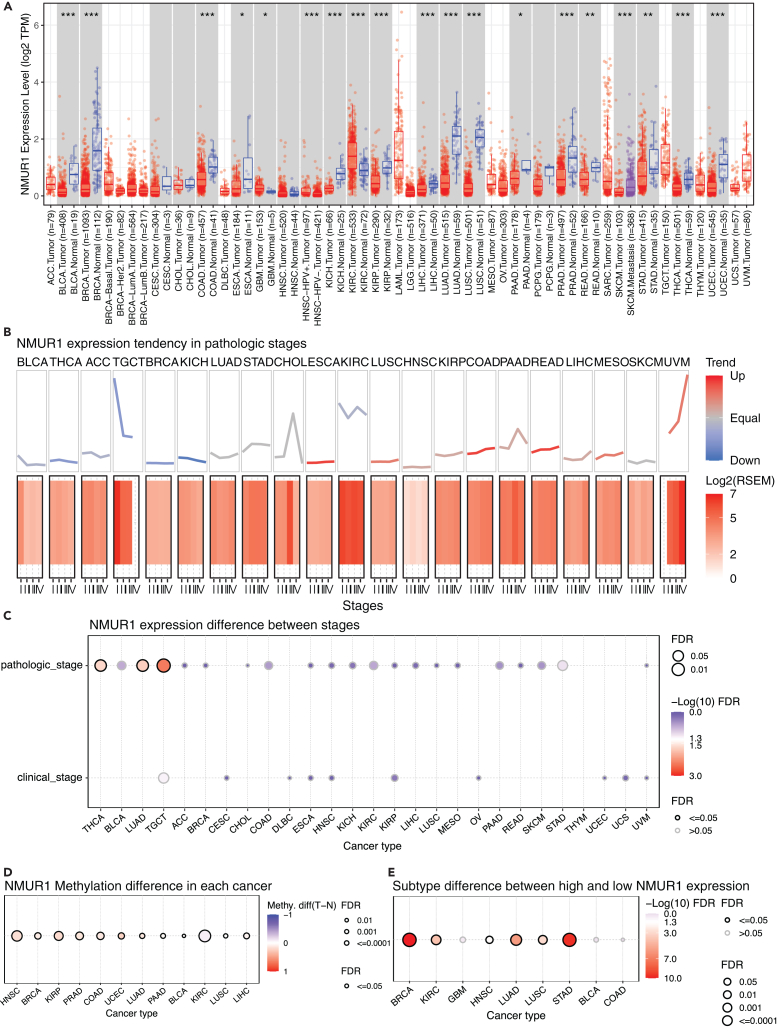
Expression signature of NMUR1 in pan-cancer (A) NMUR1 expression in tumor and adjacent normal tissues across various cancers from the TIMER2 database. Red represents tumor tissue; blue represents normal tissue; gray columns represent normal data are available. The *p* value was calculated using the Wilcoxon test. (B) NMUR1 expression trends across pathologic stages of pan-cancer based on the GSCA database. (C) Association between NMUR1 expression and stage levels in pathologic and clinical stages. (D) NMUR1 methylation differences in each cancer compared to adjacent tissue.(E) Molecular subtypes differences between high and low NMUR1 expression in pan-cancer. The bubble plot presents the FDR through bubble color and size. The row is the gene set symbol and the column is the selected cancer types. The bubble color from white to red represents the significance of FDR; the bubble size is positively correlated with the significance. The black outline border of bubbles indicates FDR ≤0.05. ∗*p* < 0.05; ∗∗*p* < 0.01; ∗∗∗*p* < 0.001. Adrenocortical carcinoma (ACC), Bladder urothelial carcinoma (BLCA), Breast invasive carcinoma (BRCA), Cervical squamous cell carcinoma and endocervical adenocarcinoma (CESC), Cholangiocarcinoma (CHOL), Colon adenocarcinoma (COAD), Lymphoid Neoplasm Diffuse Large B-cell Lymphoma (DLBC), Esophageal carcinoma (ESCA), Glioblastoma multiforme (GBM), Head and Neck squamous cell carcinoma (HNSC), Kidney Chromophobe (KICH), Kidney renal clear cell carcinoma (KIRC), Kidney renal papillary cell carcinoma (KIRP), Acute Myeloid Leukemia (LAML), Brain Lower Grade Glioma (LGG), Liver Hepatocellular Carcinoma (LIHC), Lung adenocarcinoma (LUAD), Lung squamous cell carcinoma (LUSC), Mesothelioma (MESO), Ovarian serous cystadenocarcinoma (OV), Pancreatic adenocarcinoma (PAAD), Pheochromocytoma and Paraganglioma (PCPG), Prostate adenocarcinoma (PRAD), Rectum adenocarcinoma (READ), Sarcoma (SARC), Skin Cutaneous Melanoma (SKCM), Stomach adenocarcinoma (STAD), Testicular Germ Cell Tumors (TCGT), Thyroid carcinoma (THCA), Thymoma (THYM), Uterine Corpus Endometrial Carcinoma (UCEC), Uterine Carcinosarcoma (UCS), Uveal Melanoma (UVM). See also Figure S1.

In our investigation into NMUR1’s role in oncogenesis, we explored the TIMER2 database to examine its expression levels across a spectrum of malignancies. Our analysis identified a pervasive pattern of NMUR1 downregulation in a variety of cancer types, including Bladder urothelial carcinoma (BLCA), Breast invasive carcinoma (BRCA), Colon adenocarcinoma (COAD), Esophageal carcinoma (ESCA), Glioblastoma multiforme (GBM), Head and Neck squamous cell carcinoma-HPVneg (HNSC-HPV-), Kidney chromophobe (KICH), Kidney renal clear cell carcinoma (KIRC), Kidney renal papillary cell carcinoma (KIRP), Liver hepatocellular carcinoma (LIHC), Lung adenocarcinoma (LUAD), Lung squamous cell carcinoma (LUSC), Pancreatic adenocarcinoma (PAAD), Prostate adenocarcinoma (PRAD), Rectum adenocarcinoma (READ), Skin Cutaneous Melanoma (SKCM), Stomach adenocarcinoma (STAD), Thyroid carcinoma (THCA), and Uterine Corpus Endometrial Carcinoma (UCEC) (Figure 1A). Further examination revealed that NMUR1 expression consistently varied with cancer progression, with discernible trends across different pathological stages (Figure 1B). Significantly, NMUR1’s expression was found to correlate with both pathological and clinical stages in THCA, LUAD, TGCT, and COAD, as corroborated by data from GSCA and TISIDB databases (Figures 1C and S1A). In consonance with its expression patterns in most cancers, NMUR1 demonstrated heightened methylation levels in HNSC, BRCA, KIRP, PRAD, COAD, and UCEC (Figure 1D). Further exploration of NMUR1’s correlation with molecular subtypes revealed marked subtype-specific signatures, particularly in BRCA, KIRC, LUAD, LUSC, and STAD according to the GSCA database (Figure 1E), and in BRCA, COAD, ESCA, LUSC, OV, PCPG, STAD, and UCEC based on the TISIDB database (Figure S1B). Therefore, the abnormal NMUR1 expression across pan-cancer in comparison to normal tissues implies its potential role in tumor initiation.

### Prognostic analysis of neuromedin U receptor 1 expression in human pan-cancer

**Figure 2:**
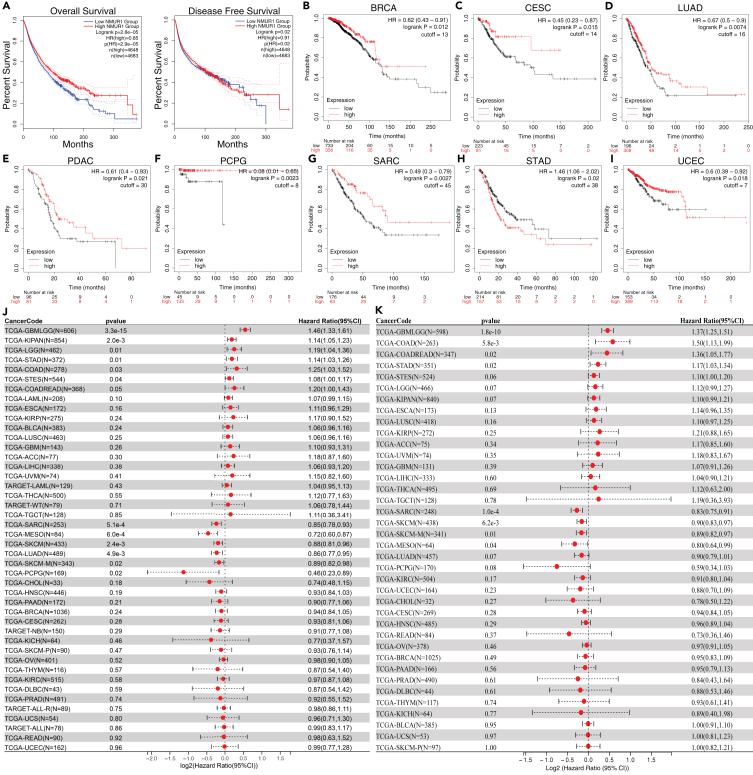
Prognosis signature of NMUR1 in pan-cancer (A) Kaplan–Meier analysis of overall survival (OS) and disease-free survival (DFS) analyses in the pan-cancer cohort from the GEPIA2 database. (B–I) NMUR1’s prognostic significance demonstrates through Kaplan–Meier analysis for various cancers. (J) Cox analysis of OS across tumors based on TCGA database. (K) Cox analysis of disease-specific survival (DSS) across tumors based on TCGA database. See also Figure S2.

Subsequent investigations included a thorough assessment of overall survival (OS) and disease-free survival (DFS) across pan-cancer cohorts to explore the potential of NMUR1 as a prognostic biomarker. The aggregate data from 33 cancer types in the TCGA dataset compellingly indicated that higher NMUR1 expression is associated with improved OS and DFS (Figure 2A). Kaplan-Meier plotter analyses^48^ further reinforced the link between NMUR1 overexpression and enhanced OS for cancers, such as BRCA, Cervical squamous cell carcinoma (CESC), LUAD, Pancreatic ductal adenocarcinoma (PDAC), Pheochromocytoma and paraganglioma (PCPG), Sarcoma (SARC), and UCEC (Figures 2B–2I and S2). Moreover, Cox proportional hazards analyses revealed that lower NMUR1 expression is significantly related to poor patient prognoses in SARC (*p* = 2.2e-4), SKCM (*p* = 5.3e-4), Mesothelioma (MESO) (*p* = 6.0E-4), Metastatic Skin Cutaneous Melanoma (SKCM-M) (*p* = 9.5e-3), LUAD (*p* = 0.01), and PCPG (*p* = 0.02) (Figure 2J). A parallel trend was observed in disease-specific survival (DSS) metrics, with reduced NMUR1 expression aligning with adverse outcomes, notably in SARC (*p* = 1.0e-4), SKCM (*p* = 6.2e-3), and MESO (*p* = 0.04) (Figure 2K). Thus, NMUR1 emerges as a prognostic biomarker with potential implications for various malignancies.

### Relationship between neuromedin U receptor 1 expression and immune infiltration in tumor microenvironment

**Figure 3:**
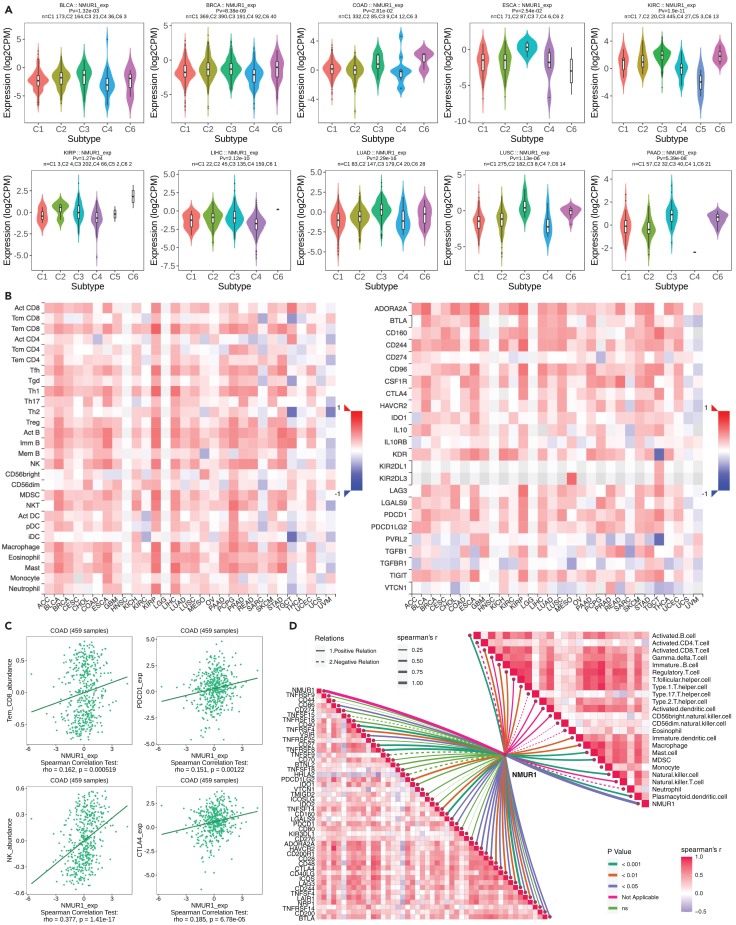
Association of NMUR1 with the immune microenvironment in various cancers (A) *NMUR1* expression levels across different immune subtypes within BLCA, BRCA, COAD, ESCA, KIRC, KIRP, LIHC, LUAD, LUSC, and PAAD. (C1: wound healing, C2: IFN-gamma dominant, C3: inflammatory, C4: lymphocyte depleted, C5: immunologically quiet, and C6: TGF-β dominant). The *p* value was calculated using the Kruskal-Wallis test. (B) Heatmap illustrating correlations between *NMUR1* expression, immune cell populations, and immune inhibitors across multiple cancer types. The color represents Spearman correlations between the abundance of tumor-infiltrating lymphocytes (left) or immune inhibitors (right) and the expression of *NMUR1*. (C) Positive correlation of *NMUR1* expression with memory CD8 T cells (Tem_CD8), natural killer (NK) cells, PDCD1, and CTLA4 in TCGA-COAD. The *p* value was calculated using the Spearman correlation test. (D) Validation of the association between *NMUR1* expression, immune modulators, and immune cells using bulk RNA-seq data from patients with CRC. The *p* value was calculated using the Spearman correlation test. See also Figures S3–S5.

After establishing the expression and survival landscape of NMUR1 in pan-cancer, we explored the immune infiltration of NMUR1. Initial investigations revealed a pronounced positive correlation between *NMUR1* and several immune subtypes (C1: wound healing; C2: IFN-gamma dominant; C3: inflammatory; C4: lymphocyte depleted; C5: immunologically quiet; C6: TGF-β dominant) in pan-cancer contexts, including BLCA (*p* = 1.32e-03), BRCA (*p* = 8.38e-09), COAD (*p* = 2.81e-02), ESCA (*p* = 2.54e-02), KIRC (*p* = 1.9e-11), KIRP (*p* = 1.27e-04), LIHC (*p* = 2.12e-10), LUAD (*p* = 2.29e-16), LUSC (*p* = 1.13e-06), and PAAD (*p* = 5.39e-06) (Figures 3A and S3). Further, *NMUR1* was positively associated with a spectrum of immune cells, notably CD8^+^ T cells and NK cells, and immune inhibitors such as PDCD1 and CTLA4, across diverse cancers (Figure 3B). These associations were robustly supported by analyses using TIMER and xCELL databases (Figures S4A and S4B). These findings suggest that NMUR1 might play a significant role in immune response across a wide range of cancer types. Given the high expression of NMUR1 in the gastrointestinal tract,^1^ we decided to focus our study on CRC. To substantiate our findings, we conducted a Spearman correlation analysis on the TCGA-COAD dataset, confirming a significant link between NMUR1 and memory CD8 T (Tem_CD8) cells (*p* = 0.000519), Natural killer (NK) cells (*p* = 1.41e-17), PDCD1 (*p* = 0.00122), and CTLA4 (*p* = 6.78e-05) (Figure 3C). Further exploration of TCGA-COAD data unveiled heightened *NMUR1* expression in most immune cells (Figure S4C). Intriguingly, elevated *NMUR1* expression corresponded with higher stromal, immune, and estimate scores (Figures S4D and S4E). Through meticulous analysis involving the ssGSEA algorithm applied to the TCGA-COAD dataset, we deciphered the intricate relationship between NMUR1 expression and various cancer related pathways. Notably, our investigation unveiled a strikingly positive association between NMUR1 and pivotal pathways implicated in tumor inflammation, Epithelial-Mesenchymal Transition (EMT), Extracellular Matrix (ECM) dynamics including degradation, angiogenesis, apoptosis, PI3K/AKT signaling, Transforming Growth Factor β (TGFB) signaling, and collagen formation. Conversely, a contrasting negative correlation was identified in pathways governing cellular response to G2M checkpoint, hypoxia, tumor proliferation, MYC targets, DNA repair, and DNA replication (Figure S5). These observations provide compelling insights into the multifaceted impact of NMUR1 on diverse molecular pathways within the context of COAD. Finally, RNA-Seq analysis within the Xiangya CRC validation cohort corroborated these findings, revealing consistent positive correlations between *NMUR1* and various immune cell subtypes, such as CD8^+^ T cells and NK cells, as well as immune checkpoints such as CD244, LAG3, CD40, and PDCD1 (Figure 3D). Collectively, our data indicate that NMUR1 is involved in shaping the tumor microenvironment and modulating immune infiltration, establishing it as a promising biomarker for predicting the efficacy of immunotherapy.

### Neuromedin U receptor 1 expression landscape in colorectal cancer single cell sequencing datasets

**Figure 4:**
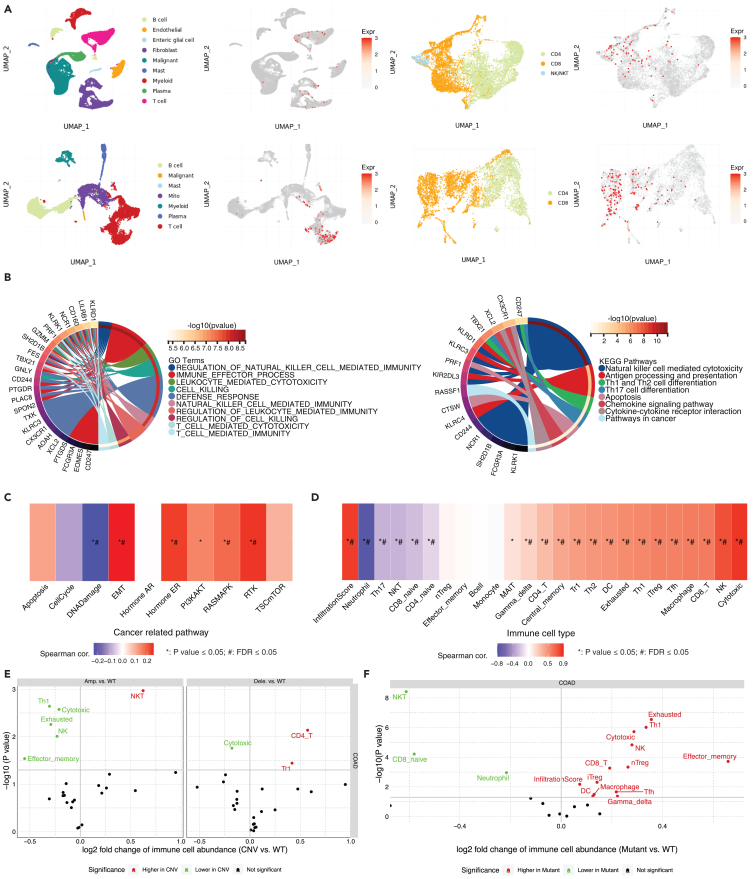
Characterization of NMUR1 expression in immune cells using single-cell RNA-seq data from CRC (A) Elevated *NMUR1* expression in CD8 T cells as observed in two independent CRC single-cell datasets. (B) Gene Ontology (GO) enrichment analysis for *NMUR1* and the top 50 correlated genes (left panel) alongside Kyoto Encyclopedia of Genes and Genomes (KEGG) pathway enrichment analysis (right panel) in two CRC single-cell datasets. (C) Correlations between cancer-related pathways and gene sets associated with *NMUR1* expression. The *p* value was calculated using the Spearman correlation test. (D) Links between gene sets related to *NMUR1* and the degree of immune cell infiltration. The *p* value was calculated using the Spearman correlation test. (E) Influence of copy number variations (CNV) in NMUR1-associated gene sets on immune cell infiltration. The *p* value was calculated using the Wilcoxon test. (F) Variation in immune infiltration levels in relation to single nucleotide variants (SNV) within *NMUR1*-associated gene sets. The *p* value was calculated using the Wilcoxon test. ∗*p* < 0.05, #FDR<0.05. See also Figure S6.

Building upon the established correlation between NMUR1 expression and the immune landscape of CRC, we further investigated NMUR1’s specific role within immune cells. Utilizing publicly available single-cell sequencing datasets, we observed a marked enrichment of NMUR1 within CD8^+^ T cells, aligning with our preliminary findings in Figures 3B and 3D (Figure 4A). This enrichment was corroborated by an extensive validation across eight CRC single-cell datasets within the TISIH database, which confirmed the heightened expression of *NMUR1* in both CD8^+^ T cells and NK cells (Figure S6A). Subsequently, we delved into the top 50 genes exhibiting correlation across two single-cell datasets (CRC_GSE108989 and CRC_GSE146771_Smartseq2), depicted as an informative heatmap (Figure S6B). Through Gene Set Enrichment Analysis (GSEA), we determined that these genes were negatively enriched in COAD samples (Figure S6C). The application of Gene Set Variation Analysis (GSVA) enabled us to quantify the collective expression of these NMUR1-correlated genes, revealing that higher GSVA scores were indicative of a decreased mortality risk (Figure S6D) and were associated with earlier pathologic stages of COAD (Figure S6E). These scores were also found to be elevated in normal tissues (Figure S6F), earlier stages of COAD (Figure S6G), and the microsatellite instability-high (MSI-H) subtype (Figure S6H). Further examination through Gene Ontology (GO) and Kyoto Encyclopedia of Genes and Genomes (KEGG) pathway analyses highlighted a significant enrichment of NMUR1-correlated gene sets in biological processes and pathways integral to NK cell-mediated and T cell-mediated immunity (Figure 4B). Positive correlations were also identified between these gene sets and pivotal cancer-related pathways, including EMT, hormone ER, RAS/MAPK, and RTK signaling pathways (Figure 4C), as well as immune infiltration scores (Figure 4D). Notably, we observed a compelling link between the levels of copy number variations (CNVs) and single nucleotide variants (SNVs) in NMUR1-correlated gene sets and the extent of immune infiltration (Figures 4E and 4F). Together, these comprehensive data shed light on the profound role of NMUR1 within the CRC immune microenvironment and underscore its functional significance.

### High neuromedin U receptor 1 expression in CD8^+^ T cell correlated with a better immunotherapy response in patients with colorectal cancer patients

**Figure 5:**
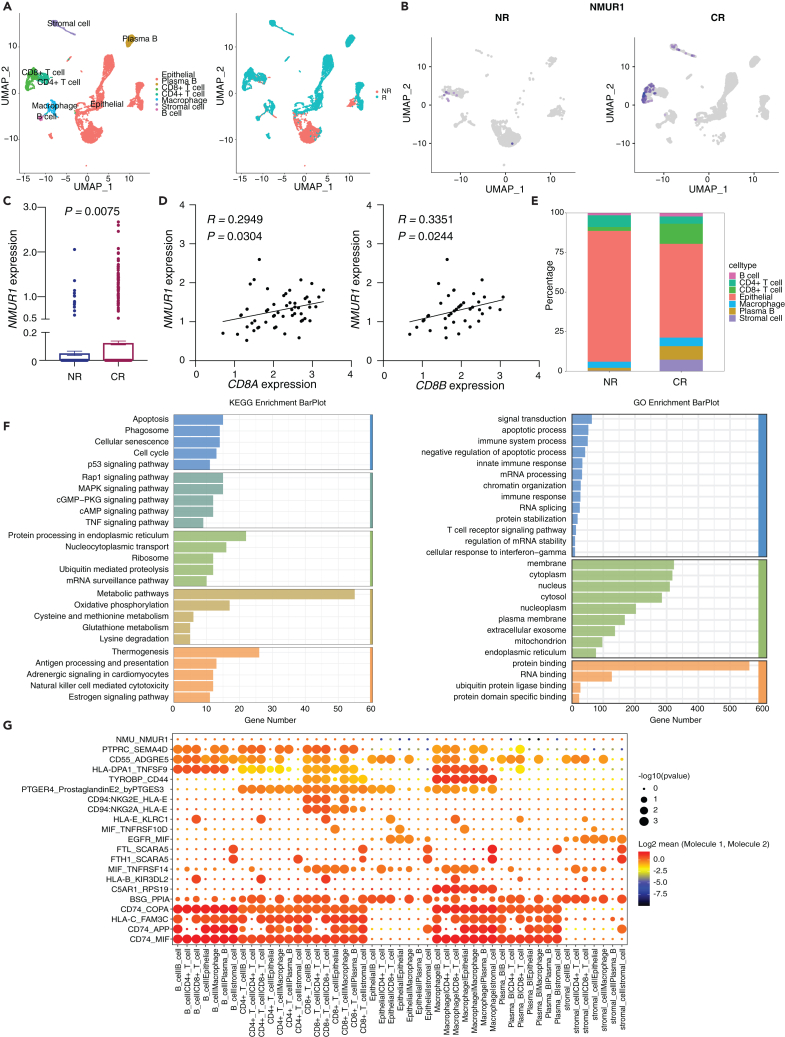
*NMUR1* expression and its correlation with immunotherapy response in patients with CRC (A) Cell type classification using UMAP visualization. (B) UMAP of *NMUR1* expression levels between non-responders (NR) and complete responders (CR) to immunotherapy. (C) Histogram of *NMUR1* expression levels between NR and CR. The *p* value was calculated using Student’s t test. (D) The relationship between *NMUR1* and *CD8A* (left panel) and *CD8B* (right panel) expression. The *p* value was calculated using the Spearman correlation test. (E) Proportional representation of different cell types in NR and CR patient. (F) KEGG pathway enrichment analysis represented as a bar plot, based on genes differentially expressed in T cells between NR and CR groups (left panel). GO enrichment analysis is depicted as a bar plot, based on differentially expressed genes in T cells between NR and CR groups (right panel). (G) Dot plots illustrate the communication patterns between receptor and ligand molecules. Data are represented as mean with SEM. See also Figures S7–S9.

In probing the involvement of NMUR1 in the COAD immune microenvironment, our study made a significant advance by analyzing single-cell sequencing data from two patients with CRC post-immunotherapy—one non-responder (NR) and one complete responder (CR). Utilizing the SciBet platform for precise cell type identification,^49^ we were able to categorize the diverse cell populations present in the samples (Figure 5A). Notably, *NMUR1* expression was significantly upregulated in the sample from the responding patient, underscoring its potential role in mediating immunotherapy efficacy (Figures 5B and 5C). Additionally, *NMUR1* showed a positive correlation with CD8^+^ T cell markers *CD8A* and *CD8B* (Figure 5D). Furthermore, a striking enrichment of CD8^+^ T cells, which are known to express NMUR1, was observed in the responding patient’s sample (Figures 5E and S7). In a deeper investigation into the functional implications of NMUR1 within T cells from NR and CR samples, we conducted a thorough GO enrichment and KEGG pathway analysis, revealing significant insights (Figures 5E and 5F). Additionally, regarding the role of NMUR1 as a receptor for NMU, we examined cellular communication at the single-cell level. Here, the NMU-NMUR1 interaction appears as a weak communicative pathway among immune cells within the CRC immune microenvironment, possibly due to the low secretion of NMU in immune cells (Figure 5G). Our finding was further supported by validation in two additional public single-cell RNA datasets, GSE205506^50^ (Figure S8) and GSE222300^51^ (Figure S9), where CD8^+^ T cell infiltration and NMUR1 enrichment were consistently observed in patients with CR. Collectively, these analyses across three independent datasets solidify the concept of NMUR1 as a determinant of immunotherapy response in CRC, enriching our understanding of its role in the immune landscape of cancer therapy.

### Neuromedin U receptor 1 as a prospective immunotherapy biomarker for colorectal cancer patient stratification

**Figure 6:**
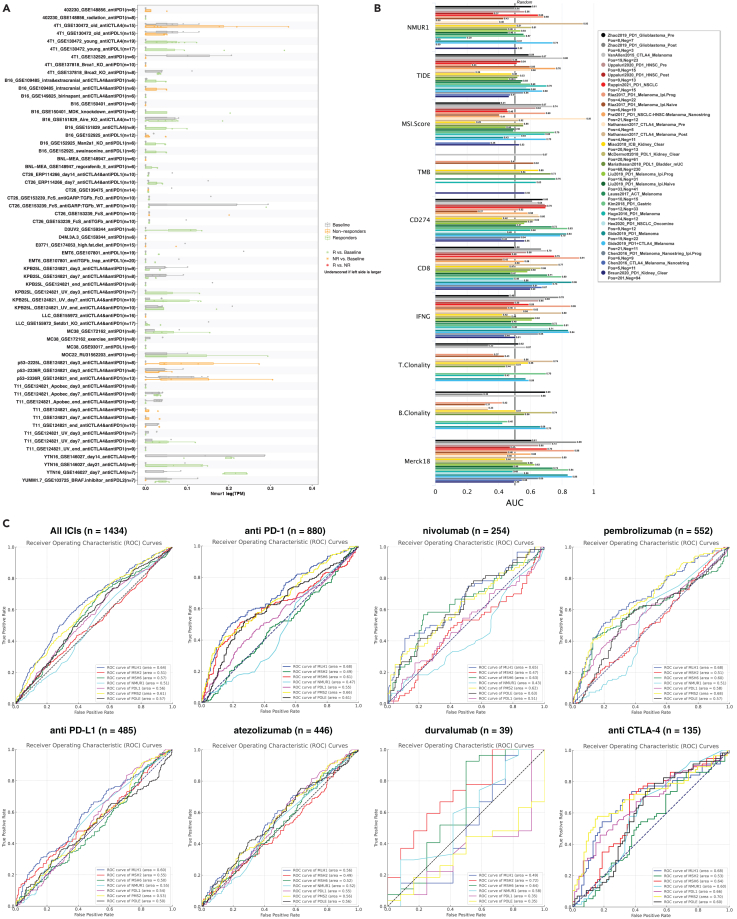
Evaluating NMUR1 as a biomarker for immunotherapy response (A) Induction of *NMUR1* gene expression across various immunotherapy models following different immune checkpoint blockade (ICB) treatments. (B) Assessment of NMUR1’s predictive capability for the immune response against a panel of established biomarkers, utilizing the TIDE platform to calculate the area under the receiver operating characteristic curve (AUC). (C) Comparative analysis of NMUR1 and other biomarkers in predicting immune response, with AUC values derived from the ROCplotter platform. See also Figure S10.

Given our insightful exploration into NMUR1’s enrichment in patients with CRC immunotherapy responder, we extended our analysis to assess its biomarker potential in publicly available immunotherapy cohorts. Utilizing the TISMO database, we conducted a comprehensive analysis of gene expression, pathways, and immune cell infiltration associated with ICB treatment. This analysis yielded a predictive model for immunotherapeutic response, revealing that NMUR1 expression is modulated by distinct ICB treatments across different cancer models (Figure 6A). The Receiver Operating Characteristic (ROC) curve, which plots the true positive rate against the false positive rate at various threshold settings, was employed as a key metric. The Area Under the ROC Curve (AUC) is particularly telling, serving as a measure of the predictive accuracy. Our results are promising, showing that NMUR1 alone achieved an AUC greater than 0.7 in two immunotherapy cohorts (Figure 6B), a performance comparable to well-established biomarkers such as TIDE, MSI score, Tumor Mutational Burden (TMB), and T and B Cell Clonality. When juxtaposed with biomarkers such as CD8, Interferon Gamma (IFNG), and the T cell-inflamed signature (Merck 18), which displayed AUCs greater than 0.7 in multiple cohorts, NMUR1 presented a modest predictive capacity. This could be attributed to the limited dataset sizes available for CRC cohorts. PD-L1, POLE, and MSI-H status are well recognized biomarkers for the guidance of immunotherapy.^47^ We then analyzed the immunotherapy cohorts from pan-cancer to compare NMUR1 with those biomarkers in different ICB treatments (Figure 6C). Furthermore, validation in the GSE235919 dataset, comprising 23 responders and 11 non-responders to CRC immunotherapy, demonstrated that higher NMUR1 expression was significantly associated with increased CD8^+^ T cell infiltration as determined by the xCELL algorithm (Figure S10A). Moreover, NMUR1 expression was consistently elevated in responders, even though without statistical significance (Figure S10B). In a comparative evaluation of biomarker potential using AUC, NMUR1 (AUC = 0.626) outperformed PD-L1 (AUC = 0.555), underscoring its viability as a biomarker in CRC immunotherapy (Figure S10C–I). In summary, these results collectively underscore the potential of NMUR1 as a significant biomarker in the realm of CRC immunotherapy.

### Deletion of neuromedin U receptor 1 potentiates the proliferation and invasion of colorectal cancer cells

**Figure 7:**
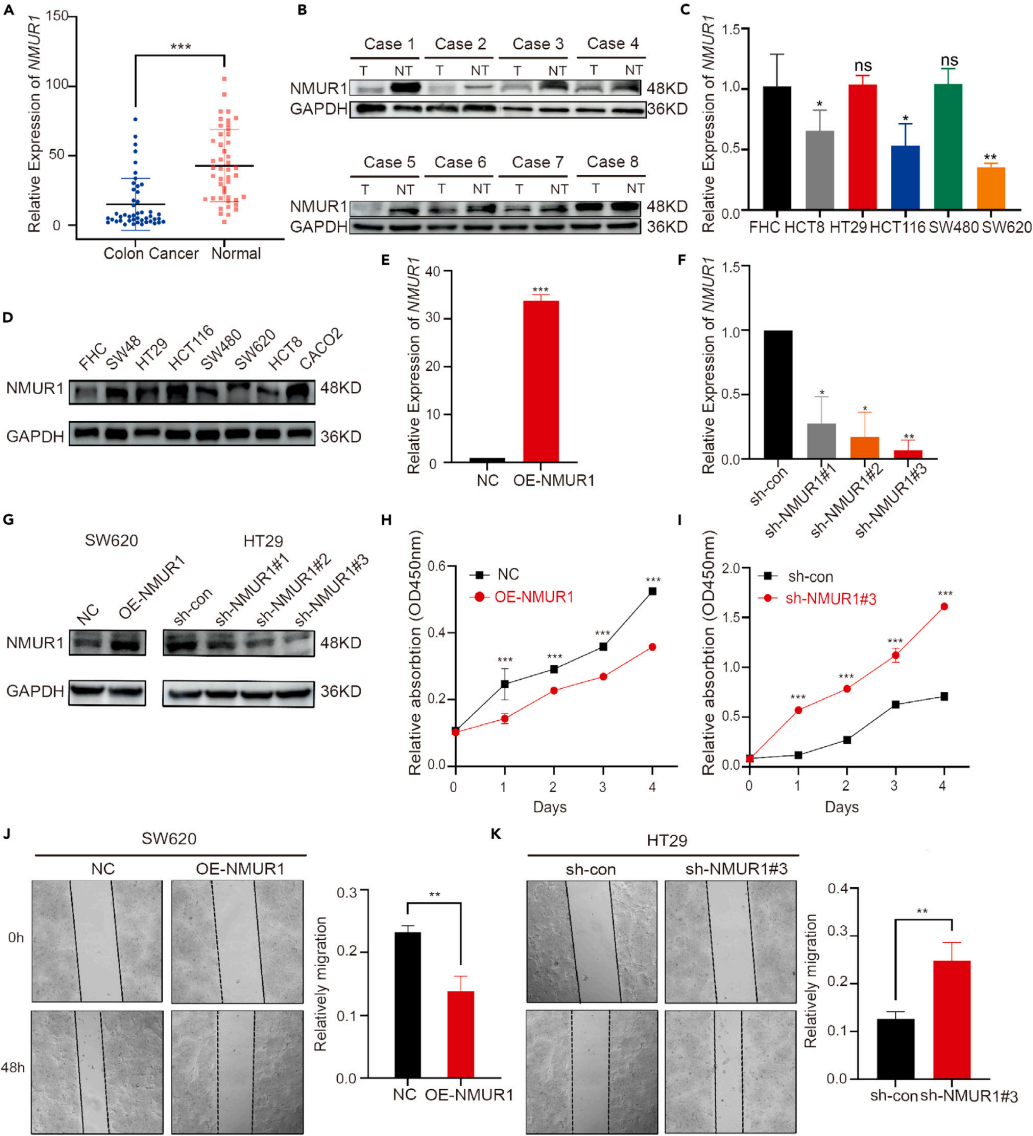
The function of NMUR1 in CRC cell lines (A) *NMUR1* mRNA expression in paired CRC patient tissues versus adjacent normal tissues, quantified by qRT-PCR (*n* = 46). The *p* value was calculated using Student’s *t*-test. (B) NMUR1 protein levels in 8 paired CRC tumor and adjacent normal tissues, analyzed by Western blot. (C) *NMUR1* mRNA expression across 5 human CRC cell lines compared to normal intestinal mucosal epithelial cells, determined by qRT-PCR. The *p* value was calculated using the ANOVA test. (D) Relative NMUR1 protein expression in 7 human CRC cell lines versus normal intestinal mucosal epithelial cells, assessed by Western blot. (E–G) Transfection efficiency of NMUR1 overexpression (OE-NMUR1) in SW620 and knockdown (sh-NMUR1#1, #2, #3) in HT29 cells, validated by qRT-PCR and Western blot. The *p* value was calculated using Student’s *t*-test in (E) and ANOVA test in (F). (H and I) Cell viability of SW620 and HT29 cells post-transfection, evaluated using CCK8 assays. The *p* value was calculated using Student’s *t*-test. (J and K) Migration ability of SW620 and HT29 cells post-transfection, assessed by wound healing assays. The *p* value was calculated using Student’s *t*-test. Data are represented as mean with SEM. Ns, not significant; ∗*p* < 0.05; ∗∗*p* < 0.01; ∗∗∗*p* < 0.001.

Analysis of total RNA from 46 CRC patient samples using RT-qPCR revealed a significant reduction of *NMUR1* mRNA expression in tumor tissues compared to adjacent non-tumor tissues (Figure 7A). Further validation was performed using protein extracts from 8 pairs of CRC tissues and adjacent non-tumor tissues. Western blot analysis confirmed the reduced expression of NMUR1 in CRC tissues, aligning with our mRNA expression findings (Figure 7B). The disparity in NMUR1 expression suggests its potential involvement in the pathobiology of CRC cells. Subsequently, we examined the NMUR1 expression signature in five CRC cell lines alongside a normal intestinal mucosal epithelial cell line through qRT-PCR and western blotting. Most CRC cell lines demonstrated lower NMUR1 expression compared to the normal cell line (Figures 7C and 7D). HT29 cells, which exhibited relatively higher levels of NMUR1, were transfected with knockdown plasmids, while SW620 cells, with lower NMUR1 levels, were transfected with overexpression plasmids. The efficiency of these transfections was confirmed by RT-qPCR and western blot (Figures 7E–7G). To discern the impact of NMUR1 on CRC cell proliferation and metastatic potential, we employed the CCK8 assay, revealing that NMUR1 overexpression markedly decreased SW620 cell viability, whereas NMUR1 knockdown increased HT29 cell viability (Figures 7H and 7I). A wound healing assay indicated that NMUR1 overexpression significantly impeded the invasive capability of SW620 cells, whereas NMUR1 knockdown had the converse effect in HT29 cells. The differences in invasion between the overexpression and knockdown groups were statistically significant (Figures 7J and 7K). In conclusion, our investigations provide substantial evidence that NMUR1 may act as a tumor suppressor in CRC.

### Neuromedin U receptor 1 prognostic value in Xiangya colorectal cancer cohort

**Figure 8:**
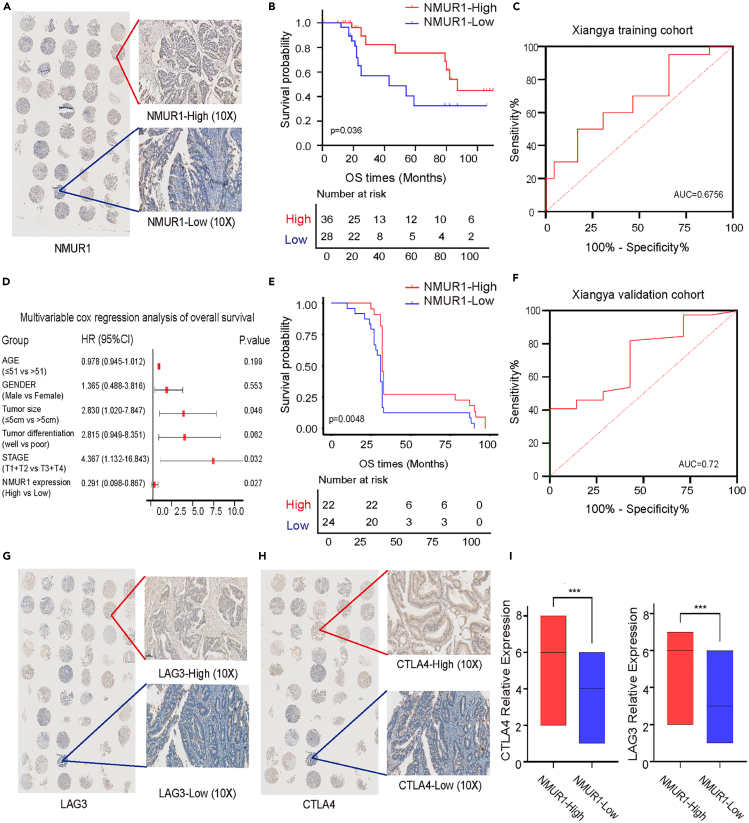
NMUR1 expression and correlation with immune markers in a clinical cohort (A) NMUR1 expression assessed by immunohistochemistry in the Xiangya training cohort. (B) Overall survival (OS) stratified by NMUR1 expression levels in the Xiangya training cohort (*n* = 64, Log rank test). (C) Area under the curve (AUC) for survival prediction based on NMUR1 expression in the Xiangya training cohort. (D) Multivariable Cox regression analysis of OS in the Xiangya training cohort. (E) OS stratified by NMUR1 expression in the Xiangya validation cohort (*n* = 46, Log rank test). (F) AUC for survival prediction in the Xiangya validation cohort. (G and H) Immunohistochemical staining of LAG3 and CTLA4 in the Xiangya training cohort. (I) Correlation between NMUR1 expression and LAG3, CTLA4 levels (*n* = 64, Student’s t-test). Data are represented as mean with SEM. ∗∗∗*p* < 0.001.

In line with previous observations, NMUR1 expression levels in tissue samples were markedly higher in tumor tissues than in adjacent non-tumor tissues. To confirm NMUR1’s expression characteristics and prognostic value, we analyzed two independent real-world cohorts (Xiangya training cohort and Xiangya validation cohort), both of which echoed the findings from our bioinformatics analysis. Tissue microarrays from 64 patients with CRC in the Xiangya training cohort were used for immunohistochemical analysis to investigate NMUR1’s prognostic significance. Patients were stratified into high (36 patients) and low (28 patients) NMUR1 expression groups, with representative staining patterns depicted in Figure 8A. Log rank test results revealed that patients with lower NMUR1 expression had a significantly poorer overall survival (OS) compared to their higher-expression counterparts, corroborating our database analysis (Figure 8B). The prognostic accuracy, as indicated by AUC for survival rate, was 0.6756 (Figure 8C). Further, multivariate Cox regression analysis identified NMUR1 expression, tumor size, and clinical stage as independent predictors of OS (Figure 8D; Table 1). Additionally, qRT-PCR of 46 CRC patient tissues from the Xiangya validation cohort further supported these findings. Kaplan-Meier analysis showed that patients with high NMUR1 expression had a better OS compared to those with low expression (Figure 8E), with an impressive AUC of 0.72 for the survival rate (Figure 8F).

**Table 1:** Cox regression analysis of overall survival in the Xiangya CRC training cohort

Variables	subgroup	Patients	Univariate analysis			Multivariate analysis		
			HR	95% CI	*p* Value	HR	95% CI	*p* Value
Age	≤50	11	0.986	(0.957–1.015)	0.343	0.978	(0.945–1.012)	0.199
	>50	53	1.000			1.000		
Gender	Male	40	1.209	(0.493–2.963)	0.678	1.365	(0.488–3.816)	0.553
	Female	24	1.000			1.000		
Tumor size	≤5cm	37	2.451	(0.999–6.011)	0.050	2.830	(1.020–7.847)	0.046
	>5cm	27	1.000			1.000		
Tumor differentiation	well	40	3.018	(1.152–7.904)	0.025	2.815	(0.949–8.351)	0.062
	poor	24	1.000			1.000		
Stage	I + II	37	6.488	(1.894–22.218)	0.003	4.367	(1.132–16.843)	0.032
	III+IV	27	1.000			1.000		
NMUR1 expression	High	36	0.393	(0.159–0.975)	0.044	0.291	(0.098–0.867)	0.027
	Low	28	1.000			1.000		

We also investigated NMUR1’s predictive value regarding immunotherapy response. Immunohistochemistry was used to validate the association between NMUR1 expression and immune checkpoints LAG3 and CTLA4, which were selected based on database analysis and clinical relevance (Figures 8G and 8H). We observed a significant positive correlation between the expression levels of NMUR1, LAG3, and CTLA4 (Figure 8I). These findings suggest that patients with higher NMUR1 expression might derive greater benefit from immunotherapy, which could account for their improved prognosis. Therefore, NMUR1 shows promise as a predictive biomarker for immunotherapy response.

## Discussion

As a major receptor of NMU which was already considered as a tumor growth and/or progression marker in endometrial, renal, and breast cancers, NMUR1 could interact with NMU leading to autocrine tumor-promoting pathway activation, including intracellular Caflux, the phosphorylation of ERK1/2 kinases and hypoxia-inducible factors activation.^9^^,^^10^^,^^11^ Considering that NMUR1 was mainly expressed in peripheral tissues especially in gut and lung,^1^^,^^5^^,^^52^ NMUR1 was widely reported to be involved in airway inflammation and immune response,^15^^,^^53^ while few literatures revealed the association between NMUR1 and gastrointestinal immune response, especially in gastrointestinal cancer. Our study identified NMUR1 as a potential biomarker for CRC prognosis and immunotherapy response by combining multi-omics analyses and *in vitro* functional experiments. We proved that high NMUR1 expression contributed to the inhibition of CRC cells proliferation and invasion, and was positively correlated with immune checkpoints in patients with CRC’ tissues.

In this study, firstly, we explored the expression level of NMUR1 at pan-cancer level using TCGA and GEO databases. The results obtained reveal that NMUR1 had a significantly lower expression level in COAD, LUAD, and so forth, which is consistent with recent publications in CRC and lung cancer.^7^^,^^11^ NMUR1 expression level was also found to be a positive correlation with the different stages and molecular subtypes of certain cancers such as COAD, TGCT, BRCA, and so forth. For the NMUR1 prognostic values, we performed the analysis based on GEPIA2 and Kaplan-Meier Plotter. Similar to previous research, decreased NMUR1 expression correlated with a poor prognosis in certain cancers, including STAD, COAD, GBM, LGG, and BRCA, as well as in pan-cancer. Taken together, these pan-cancer data offered us a perspective that NMUR1 played a crucial role in the initiation and progression of tumors.

The TME has gained significant attention for its dynamic role in tumor promotion or suppression. Recent literature has categorized tumors as "cold" or "hot" based on the heterogeneity of TILs, stromal cells, and extracellular matrix components.^54^ Insight distinction and alteration of hot tumor is beneficial to the intervention of immunotherapy strategies. We conducted a bioinformatics analysis which revealed a huge difference proportion of NMUR1 in different cancers. Notably, high expression of NMUR1 in TCGA-COAD was found in the C3 (Inflammatory) subtype which presented increased expression level of Th17 and Th1 and low cancer cells proliferation when compared with lower expression in C1 (Wound Healing) and C2 (IFN-γ Dominant) subtypes which herald boost cancer cells proliferation and worse prognosis. These results further indicated that NMUR1 might be an important molecular and biomarker in different subtypes of cancer.

TILs are diverse within the TME, affecting tumor metastasis, angiogenesis, and immunotherapy resistance.^55^ CD4^+^ T cells, multiple subtypes including T helper type 1 (Th1), Th2, Th17, Th9, Treg and T follicular helper cells, contribute to diverse functions that are opposite in antitumor immunity leading to an opposite outcome.^56^ CD8^+^ T cells which were also called cytotoxic T lymphocytes (CTLs) serve as a durable and critical antitumor immune effect via binding to membrane-expressed T cell receptors (TCR) leading to the activation of apoptosis.^57^^,^^58^ NK cells, a member of the innate lymphoid cell (ILC) family, have a significant impact on anti-tumor immunity which presents direct cytolytic activity interacted with target cells, up-regulated expression of TNF and death-inducing ligands, and a capacity to secrete multiple cytokines and chemokines.^59^ Tumor-associated macrophages (TAM), macrophages infiltrated in tumor types, exerting antitumor or protumor function according to the different status, M1 (antitumor role) and M2 (protumor role). In this study, we initially discovered an association between NMUR1 expression and the proportion of immune cell infiltration in pan-cancer using TIMER2 and xCELL databases. In line with our hypothesis, NMUR1 was enriched in CD4^+^ T cells, CD8^+^ T cells, and macrophage cells across different cancers and positively connected with StromalScores, ImmuneScores, and ESTIMATE scores in pan-cancer. Based on the above results, NMUR1 showed a robust correlation with multi-types of tumor-associated immune infiltration cells.

TISCH database was selected to explore how NMUR1 affects CRC TME in single-cell insight. The results obtained show different immune cell distributions. Similar to previous results, higher NMUR1 expression was observed in CD4^+^ T cells and CD8^+^ T cells in five independent CRC datasets. Notably, Guozhong Xiao et al. found that CD4^+^ T cells in overweight/obese CRC exhibited higher expression of immune checkpoint molecules and immunosuppressive microenvironment.^60^ NMU-NMUR1 signaling has also been investigated to show a connection with the metabolism and regulation of anti-tumor activity of CD8^+^ T cells and glycolysis of tumor cells.^19^^,^^61^^,^^62^^,^^63^ Our findings indicated a pronounced correlation between increased NMUR1 levels in CD8^+^ T cells and enhanced immunotherapy outcomes, suggesting that NMU-NMUR1 signaling could potentially alter CD8^+^ T cell metabolism in the CRC microenvironment. In addition, the ratio of NK cells exhibited a positive association with NMUR1 expression in the CRC_GSE146771_10X dataset. Therefore, the patients with high expression of NMUR1 might present a tumor suppressive TME and benefit more from immunotherapy strategies. Besides, a positive correlation with chemokines and related receptors was noticed in the high expression of NMUR1. Surprisingly, the remarkable results reported that NMU-NMUR1 signaling performs a vital function in immunity and allergic inflammation by the activation of ILC2s causing increased secretion of IL-5, IL-9, and IL-13.^16^^,^^17^^,^^18^ Consistently, high expression of NMUR1 was found to correlate with the upregulated proportion of NK cell which is a member of the ILC family and secrets diverse chemokine.

TMB and MSI have been proven to increase mutation frequency which might lead to immunogenic neoantigens transcription and translation because of mismatch repair (MMR) deficiency. They serve as the predicting factors for immunotherapy efficacy.^64^ A hot tumor feature of a high T cell infiltration degree was found to be effective in ICI therapy. For instance, CTLA4, a classic inhibitory receptor expressed in exhausted or dysfunctional TILs, inhibits the early activation and differentiation of T cells. Strategies used to inhibit CTLA4 have now been approved to be effective in the treatment of advanced-stage melanoma,^65^ RCC,^66^ and NSCLC.^67^ We performed IHC to detect the relative protein expression of classic ICP genes, CTLA4, and LAG3 in the Xiangya cohort. TMB and MSI are predictors of immunotherapy efficacy, and our results suggest NMUR1 could be added to this repertoire, especially considering its positive correlation with immune checkpoint protein LAG3 and CTLA4, indicative of a prognostic and therapeutic biomarker for CRC.

NMU has been shown to play an important role in cancer through NMUR1 or NMUR2 dependent pathway. Przygodzka et al. had tested NMU mRNA expression and protein level in 6 CRC cell lines (Caco-2, HT29, SW620, HCT15, HCT116, and SW480).^11^ Even though all the tested CRC cells expressed *NMU* mRNA, the NMU protein was only detectable in the lysates and cell-conditioned media of HCT15, HCT116, and SW480 cells. And NMU induced an invasive phenotype of CRC cells in a NMUR2 dependent way instead of NMUR1. Notably, CRC cells exhibited higher expression of NMUR2 and lower expression of NMUR1. And cognate residues for both NMUR1 and NMUR2 are completely conserved, thus making highly similar interactions with peptide.^68^ Given these findings, we hypothesize that overexpression of NMUR1 might compete with NMUR2 for NMU binding, leading to a suppressive effect in CRC cells by interfering with NMUR2-dependent signaling. It is worthwhile to investigate if there is a competition between NMUR2 and NMUR1 for NMU in the future experiment.

Collectively, our study showed that downregulated expression of NMUR1 was correlated with worse prognosis and immune infiltration in CRC. NMUR1 has emerged as an effective predictor of CRC patient prognosis and immunotherapy response, highlighting its significant role in inhibiting CRC proliferation and invasion. Thus, NMUR1 represents a promising biomarker for prognosis and immunotherapy response in CRC.

### Limitations of the study

There are still some limitations need to be considered in our study. Even though we thoroughly searched the public database, there are still extremely few cohorts for CRC immunotherapy. In the future, we need to increase the sample size and demographic diversity. The association between the expression of NMUR1 and CTLA4 is conflicting between IHC experiment results in the Xiangya cohort and the bioinformatics analysis. This may be due to the limited bioinformatics database and the inaccurate assessment of the complex and heterogeneous TME using tissue microarray. The information from databases exhibited conflict and specificity deficiency and the verification of the NMUR1 expression signature was only performed in CRC tissue samples and CRC cell lines. The definite molecular mechanisms and exact pathways of how NMUR1 participates in tumor progression and invasion, and immune regulation remain are still required to be investigated in mice model and *in vitro* experiments. It has been proven that NMU may be an important resistance-enhancing factor and display an anorexigenic effect in animal models,^21^^,^^61^ deeper insight into NMUR1 as a cancer cachexia regulator and drug target would be critical in clinical significance.

## STAR★Methods

### Key resources tables

**Table:** 

REAGENT or RESOURCE	SOURCE	IDENTIFIER
**Antibodies**		

Anti-hu-NMUR1	Invitrogen	Cat. # PA5-30376; RRID: AB_2547850
Anti-GAPDH	Abcam	Cat. # ab181602; RRID: AB_2630358
Anti-hu-LAG3	CST	Cat. # 15372; RRID: AB_2798739
Anti-hu-CTLA4	Abcam	Cat. # ab237712; RRID: AB_2905652

**Biological samples**		

Human CRC and adjacent non-tumor tissues	Xiangya Hospital of Central South University	N/A

**Chemicals, peptides, and recombinant proteins**		

Trizol	Invitrogen	Cat. # 10296010
RPMI-1640 medium	HyClone	Cat. # SH30027.FS
L-15 medium	Gibco	Cat. # 21-083-027
Fetal Bovine Serum	Gibco	Cat. # 10437028
Immobilon Western HRP substrate	Millipore	Cat. # WBKLS0500

**Critical commercial assays**		

SYBR Green fluorescent-based assay	TaKaRa Bio Inc	Cat. # 638320
PrimeScript RT-PCR Kit	TaKaRa Bio Inc	Cat. # RR014B
Lipofectamine 3000	Invitrogen	Cat. #L3000008
Cell Counting Kit 8	Dojindo	Cat. # CK04

**Deposited data**		

Single cell RNA-seq data	This study	GSA-Human: HRA006722 & HRA007564
Bulk sequencing data	This study	GSA-Human: HRA007645
Public single cell RNA-seq data	GEO	GSE108989 GSE136394 GSE139555 GSE146771 GSE166555 GSE179784 GSE205506 GSE222300 GSE235919

**Experimental models: cell lines**		

Human CRC cell lines and normal intestinal epithelial cells	Institutes of Biomedical Sciences	N/A

**Oligonucleotides**		

*NMUR1*	5′-GCCGGAGACAAGTGACCAAGA-3′	5′-TGACACGACGCTCCACATG-3′
*GAPDH*	5′-CTGGGCTACACTGAGCACC-3′	5′-AAGTGGTCGTTGAGGGCAATG-3′

**Software and algorithms**		

R studio Version 1.2.1335 (R version4.1.1)	RStudio, Inc.	https://www.rstudio.com/
GraphPadPrism7.0	GraphPad	www.graphpad.com
Adobe Illustrator 2023	Adobe	http://aotucad2.xmjfg.com/pg/230.html
ImageJ	ImageJ	https://imagej.nih.gov/ij/
ImageLabsoftware	Bio-Rad	www.bio-rad.com
OmicStudio	LC-Bio Technology Co	https://www.omicstudio.cn/tool
SPSS20.0	IBM	https://spss.en.softonic.com/

### Resource availability

#### Lead contact

Further information and requests for resources and reagents should be directed to and will be fulfilled by the lead contact Dr. Shan Zeng (zengshan2000@csu.edu.cn).

#### Materials availability

This study did not generate unique reagents.

#### Data and code availability

• All the public datasets can be downloaded in the Cancer Genome Atlas (TCGA) (https://cancergenome.nih.gov/), Gene Expression Omnibus (GEO) (https://www.ncbi.nlm.nih.gov/geo/) database, and UCSC Xena (https://xena.ucsc.edu/) database. Bulk sequencing data can be found in supplementary table and are available on GSA human database (https://ngdc.cncb.ac.cn/gsa-human/) under accession number HRA007645. Single cell sequencing data are available on GSA human database under accession number HRA006722 & HRA007564. As per local regulations, human sequencing data cannot be made publicly accessible. Access can be requested by following the guidance from GSA Human database (https://ngdc.cncb.ac.cn/gsa-human/document/GSA-Human_Request_Guide_for_Users_us.pdf).^69^^,^^70^
• This paper does not report original code.
• Any additional information required to reanalyze the data reported in this paper is available from the lead contact upon request.

### Experimental model and study participant details

#### Patients and follow-up

From October 2011 to November 2019, clinical samples were gathered from patients diagnosed with CRC at Xiangya Hospital of Central South University. In the Xiangya cohort, a total of 110 patients were recruited for the retrospective study. Follow-up was completed in November 2021. Detailed information of CRC patients was acquired from the Xiangya digital medical records system. Overall survival (OS) was chosen as the endpoint of this study. We separated 110 patients into a training cohort (64 patients) and a validation cohort (46 patients). This study was reviewed and approved by the Xiangya Hospital Medical Ethics Committee of Central South University (No.201905131), and got the consent from all participates. Sex/gender as a variable factor did not show significant difference.

#### Cell lines and cell culture

Human CRC cell lines SW480, SW620, HCT8, HT29, HCT116 and human normal intestinal epithelial cell FHC were supplied by the Institutes of Biomedical Sciences (IBS, Shanghai, China). Cell lines were cultured in RPMI-1640 medium (HyClone, Cat. # SH30027.FS, UT) or L-15 medium (Gibco, Grand Island, NY) with 10% FBS (Gibco, Grand Island, NY) and were cultured in a humidified 37°C and 5% CO_2_ atmosphere.

### Method details

#### Data collection

The Cancer Genome Atlas (TCGA) and Genotype-tissue expression (GTEx) profiles and clinical information were collected from the UCSC Xena (https://xena.ucsc.edu/) database for this investigation.^71^ R software (version 4.1.1) and related website databases were used to assist in the analysis of the data.

#### Differential expression of NMUR1 analysis

The expression differences of NMUR1 in pan-cancer were investigated using TCGA and GTEx. TISIDB (http://cis.hku.hk/TISIDB/index.php) was chosen to examine NMUR1 expression in TCGA tumor samples from various immunological subtypes and molecular subtypes.^72^ To identify expression differences between tumor and normal tissues, we employed the R "limma" package.^73^ Boxplots were generated using the R "ggplot2″ package.^74^ The methylation analysis was performed by GSCA tool, which performed correlation analyses for each gene to identify the methylation site most negatively correlated with the gene’s expression.^75^

#### Prognostic value analysis

GEPIA2 (http://gepia.cancer-pku.cn/) was elected to detect OS and DFS survival data for NMUR1 in all tumor types. Kaplan–Meier plotter (http://kmplot.com/analysis/) was utilized to evaluate the prognosis of NMUR1 expression in various cancers,^76^ and the best performing cutoff was determined as stated in Nagy et al.^48^ By using SangerBox online database,^77^ we investigated the impact of NMUR1 on patients’ survival in pan-cancer according to univariate Cox regression.

#### Assessment of immune infiltration levels

Immunedeconv, an R package that combines with TIMER, xCELL, MCP-counter, CIBERSORT, EPIC and quanTIseq algorithms, was considered to explore immune scores.^78^ To determine the Stromal, Immune and ESTIMATE scores in each sample based on NMUR1 expression, we chose the R "ESTIMATE" package.^79^ GSVA package^80^ was chosen to estimate NMUR1 immune cell infiltration level. The markers for 24 immune cells were obtained from an article in Immunity.^81^ Besides, we downloaded the pan-cancer dataset from the UCSC (https://xenabrowser.net/) database, and further extracted NMUR1 expression and immune-related genes including chemokine, receptor, MHC, Immunoinhibitor and Immunostimulator in each sample.

#### NMUR1 related gene functional and pathway enrichment investigation

We collected the gene sets contained in the relevant pathways^82^ and calculated the enrichment score of NMUR1 on each pathway according to the single sample Gene Set Enrichment Analysis (ssGSEA) algorithm, so as to obtain the relationship between NMUR1 and gene ontology (GO) biological function. By calculating the correlation between gene expression and pathway score, we can get the gene-pathway relationship. The R "GSVA" package was used to perform a Spearman correlation analysis on the relationship between NMUR1 and pathway scores.^80^^,^^82^

#### Single cell sequencing data analysis

CancerSEA (http://biocc.hrbmu.edu.cn/CancerSEA/home.jsp) is an appropriative online database utilized to investigate distinct functional states across various cancers at a single-cell level.^83^ Based on single-cell sequencing profiling, we studied the NMUR1 function characteristics in 14 different states and drew a heatmap to explore the differences in pan-cancer. TISCH (https://doi.org/10.1093/nar/gkaa1020) is a scRNA-seq atlas that provides single-cell transcriptomic profiles data collected from GEO^84^ and ArrayExpress,^85^ which was chosen for a comprehensive exploration of the TME components and its correlation of NMUR1 expression at the single-cell level.^86^ We utilized the TIGER database (http://tiger.canceromics.org/#/home) to show NMUR1 expression in two CRC single cell sequencing.^87^^,^^88^ In addition, to assess the correlation between NMUR1 and immune infiltration in colon cancer, we applied the GSCA R package to calculate the NMUR1 score, and further explored the correlation of the score with multiple immune cells, CNV, and SNV.

#### Biomarker analysis

The data comparing gene expression levels across different tumor models and ICB treatments, between pre- and post-ICB treatment and responders and non-responders were acquired from the Tumor Immune Syngeneic MOuse (TISMO, http://tismo.cistrome.org/). TIDE platform can perform the comparison between the custom biomarker and other published biomarkers based on their predictive power of response outcome and overall survival.^89^^,^^90^ ROC plotter is a platform to investigate biomarkers of immunotherapy response in a large cohort of solid tumor samples.^91^

#### Western blot

We carried out western blot to analyze the relative protein expression under standard technique. Briefly, the CRC cell (5×10^6^ cells) and CC tissue samples (40mg) were lysed by RIPA cell lysis (NCM Biotech, Suzhou, China) with protease inhibitor and phosphatase inhibitor. The proteins samples (20ug) were then loaded in a 12.5% SDS PAGE and transferred to PVDF membrane under constant 300 mA at 90 min. The membranes were incubated in NMUR1(1:1000) and GAPDH (1:10000) Rabbit Polyclonal antibody at 4°C overnight (NMUR1, #PA5-30376, Invitrogen, USA; GAPDH, ab181602, Abcam, USA). The membranes were incubated in HRP-conjugated IgG for 2 h at 4°C after being washed by TBST. The chemiluminescence substrate Immobilon (Merck Millipore, Cat#WBKLS0500, Germany) was employed for signal detection according to the manufacturer’s instructions. The ChemiDoc XRS+ System was used to automatically detect the signals (Bio-Rad, Hercules, CA). GAPDH protein expression was chosen as internal control.

#### RNA extraction and real-time quantitative PCR (RT-qPCR)

The relative mRNA levels were assessed by reverse transcription PCR (RT-PCR). Total RNA was isolated from the CRC cells and CRC tissue samples by Trizol reagent (Invitrogen, Cat. #10296010, CA), and the PrimeScript RT-PCR Kit (TaKaRa Bio Inc., Cat. # RR014B, Japan) was performed for cDNA synthesis. RT-qPCR was performed on a ViiATM7 RT-PCR system (Applied Biosystems, Carlsbad, CA) using SYBR Green fluorescent-based assay (TaKaRa Bio Inc., Cat. #638320). After normalizing to GAPDH expression level, the relative expression level of NMUR1 was determined using the 2^−(ΔΔCt)^ method. Sequences of primers:

NMUR1: forward 5′-GCCGGAGACAAGTGACCAAGA-3′; reverse 5′-TGACACGACGCTCCACATG-3′.

GAPDH: forward:5′-CTGGGCTACACTGAGCACC-3′; reverse 5′-AAGTGGTCGTTGAGGGCAATG-3′.

#### Construction of plasmid and transfection

SW620 and HT29 human colon cancer cells were selected according to their expression levels of NMUR1 and were transfected with lentivirus expressing shRNAs targeting NMUR1 and lentivirus selectively expressing NMUR1 (GeneChem Inc, Shanghai, China) by using Lipofectamine 3000 (Invitrogen, Carlsbad, CA). The target sequences of shRNAs were as follows: sh-NMUR1 #1 (5′-ccGGAGACAAGTGACCAAGAT-3′), sh-NMUR1 #2 (5′-cgCTACTGTTTGAGATGGTCT-3′), and sh-NMUR1 #3 (5′-gcGCACGCCTACCAACTACTA-3′). Cells were cultured for 72 h following transfection and selected in puromycin (3 μg/mL). Effective knockdown and overexpression were evaluated by fluorescence microscopy. RT-qPCR and western blot were conducted to verify the transcriptional and translational transfection efficiency.

#### Cell Counting Kit 8 (CCK8)

A CCK8 Kit (Dojindo, Tokyo, Japan) was used for determining cell viability. 2.0 × 10^3^/well cells were seeded and 10 μL/well CCK8 was added into 96-well plates. The plates were incubated at 37°C for 2 h and were then measured using OD 490 nm absorbance values each day.

#### Wound healing assay

Wound-healing experiments were used to determine cell invasion abilities. Briefly, HT29 and SW620 cells (1×10^6^/well) were seeded into 6-well plates. A wound was generated in the plate scratched by a 10 μL pipette tip. The floating cells were gently washed by PBS and cultured in a serum-free RPMI-1640 or L-15 medium. The wound healing status was observed under a microscope and representative fields pictures were captured at 0 and 48 h.

#### Immunohistochemistry staining (IHC)

The tissue microarray (TMA) was established from tissue samples of 64 CRC patients after follow-up. CRC tissue sample was immersed in paraformaldehyde and embedded in paraffin to constitute tissue microarray. After slicing into 4 μm slides, dimethyl benzene was used for dewaxing and ethanol was used for hydration, EDTA buffer was chosen for antigen retrieval at 95°C for 20 min 10% goat serum was used to block charged groups for 30 min. Then the slides were incubated with NMUR1 (1:100), LAG3 (1:200) and CTLA4 (1:500) Rabbit Polyclonal antibody at 4°C overnight (NMUR1, #PA5-30376, Invitrogen, USA; LAG3, #15372, CST, USA; CTLA4, ab237712, Abcam, USA). The secondary antibody was incubated for 30 min. After DAB staining, the slides were dehydrated, transparent, sealed with gumdrops and scanned by a Multispectral imaging system. IHC staining score was evaluated by 3 separate pathologists according to the percentage of positive staining cells (0–25%, 1; 26%–50%, 2; 51%–75%, 3; 76–100%, 4) and staining intensity (no staining, 0; Yellow, 1; Light brown, 2; Brown, 3). The results were sorted into two groups based on the sum of the percentage of positive cells and staining intensity scores: high expression (total score ≥4) and low expression (total score<4).

#### Single cell RNA sequencing and bulk RNA sequencing of real-world cohort

2 fresh tumor samples were collected from CRC patients received immunotherapy of Xiangya Hospital of Central South University. One patient showed no response (NR) and the other showed good response. Sequencing was performed according to the 10x Single Cell 3′ Reagent Kit. The furthering analysis was based on R software. 55 fresh tumor samples were collected from CRC patients of Xiangya Hospital of Central South University and were stored in liquid nitrogen. After TRIzol (Invitrogen, Cat. # 10296010, CA, USA) treated, total RNA was extracted from tissues samples for sequencing. Raw reads were processed using the Illumina Hiseq. Trimmomatic was utilized for quality control. Reads per kilobase per million reads (RPKM) was used for calculating gene expression levels. Bioinformatic analysis was performed using the OmicStudio tools at https://www.omicstudio.cn/tool.^92^

### Quantification and statistical analysis

Data processing, statistical analysis, and visualization were performed in R software (version 4.1.1), SPSS 23, and GraphPad Prism 9 software. All results of this study were estimated for statistical significance using Wilcoxon test, Kruskal-Wallis test, Spearman coefficients, Student t-test, and ANOVA appropriately. The Kaplan-Meier method and log rank test were selected to perform survival analysis. Univariate and multivariable Cox regressions were utilized to calculate HRs and explore the predictive biomarkers that independently affected CRC prognosis. Statistical significance was defined as *p* values or adjusted *p* values less than 0.05.

## Supplemental information

Document S1. Figures S1–S10

Table S1. CRC bulk sequencing count data, related to Figure 3

Table S2. CRC patient clinical characteristics in the Xiangya cohort, related to Figure 8
