# Neuromedin U receptor 1 deletion leads to impaired immunotherapy response and high malignancy in colorectal cancer

**DOI:** 10.1016/j.isci.2026.115270

**Published:** 2026-03-11

**Authors:** Yulai Zhou, Xiangyang Zhang, Yan Gao, Yinghui Peng, Ping Liu, Yihong Chen, Cao Guo, Gongping Deng, Yanhong Ouyang, Yan Zhang, Ying Han, Changjing Cai, Hong Shen, Le Gao, Shan Zeng

## Main text

(iScience *27*, 110318; July 19, 2024)

Post-publication, a reader contacted the journal to raise concerns on data and findings presented in this manuscript. In response, the journal promptly initiated an investigation and contacted the corresponding authors for clarification. The authors acknowledged honest mistakes in the assembly of Figure 7D and provided a satisfactory explanation along with raw data. The revised version of Figure 7 is included below. The authors would like to thank the reader and apologize for any confusion or inconvenience this issue may have caused.

**Figure:**
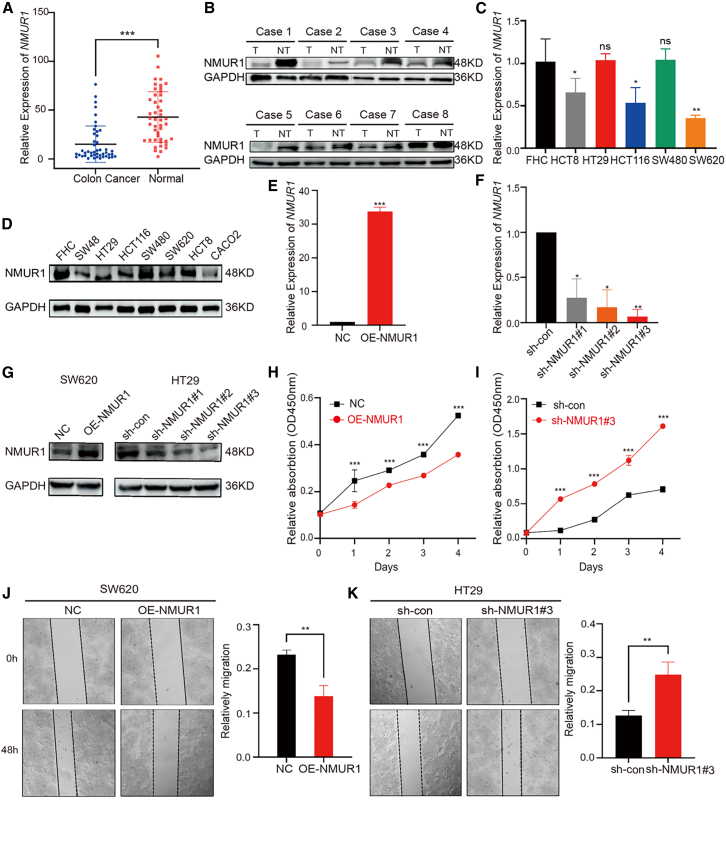
Figure 7. The function of NMUR1 in CRC cell lines

