# Supplementary material for: Neuromedin U receptor 1 deletion leads to impaired immunotherapy response and high malignancy in colorectal cancer
Source: iScience. 2024 Jun 20;27(7):110318. doi: 10.1016/j.isci.2024.110318 (PMC11269305; doi:10.1016/j.isci.2024.110318)
Supplement: Document S1. Figures S1–S10 [file mmc1.pdf]

iScience, Volume *27*

Supplemental information

Neuromedin U receptor 1 deletion leads to impaired immunotherapy response and high malignancy in colorectal cancer

Yulai Zhou, Xiangyang Zhang, Yan Gao, Yinghui Peng, Ping Liu, Yihong Chen, Cao Guo, Gongping Deng, Yanhong Ouyang, Yan Zhang, Ying Han, Changjing Cai, Hong Shen, Le Gao, and Shan Zeng

Supplemental information

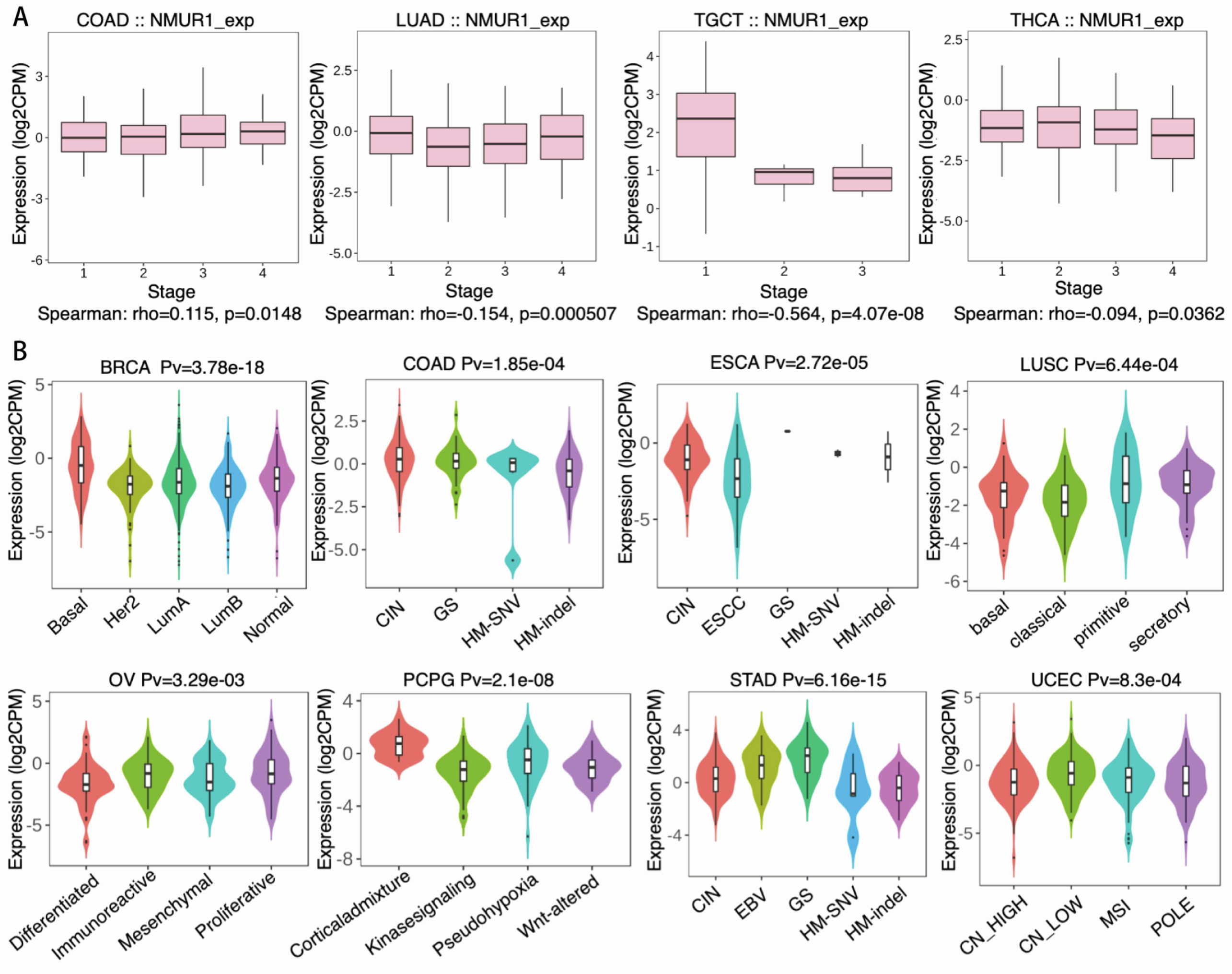

Figure S1. NMUR1 expression variance across cancer stages and subtypes, Related to Figure 1. (A) Significant correlations of NMUR1 expression with clinical stages in COAD, LUAD, TGCT, and THCA. The p value was calculated using Spearman correlation test. (B) Analysis of NMUR1 expression associations with molecular subtypes in various cancers, including BRCA, COAD, ESCA, LUSC, OV, PCPG, STAD, and UCEC, as sourced from the TISIDB database. The p value was calculated using Kruskal-Wallis test.

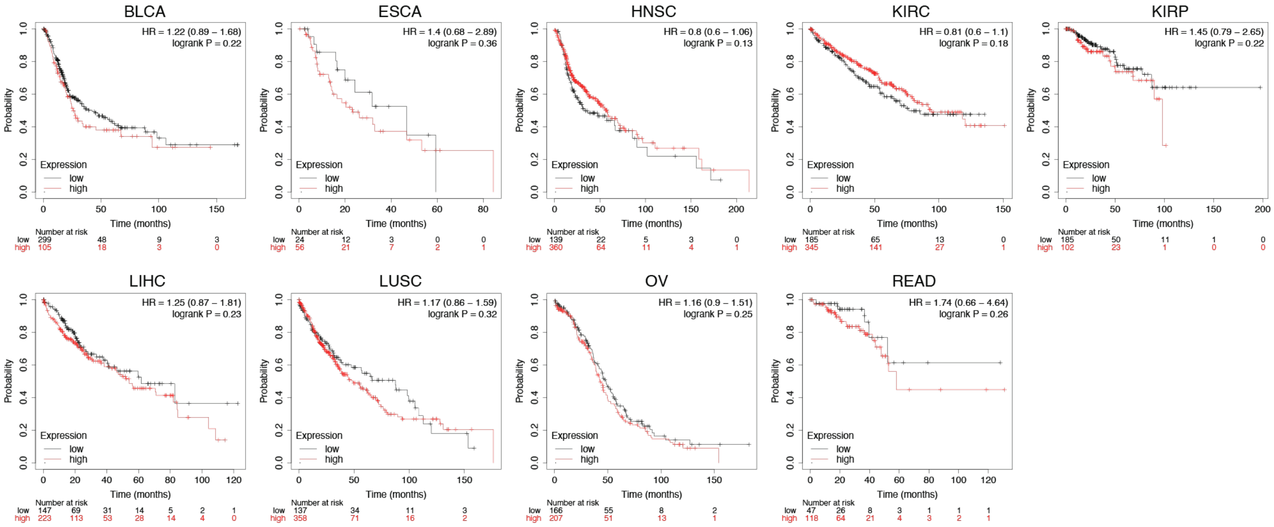

Figure S2. Prognostic significance of NMUR1 through Kaplan–Meier analysis for various cancers, Related to Figure 2.

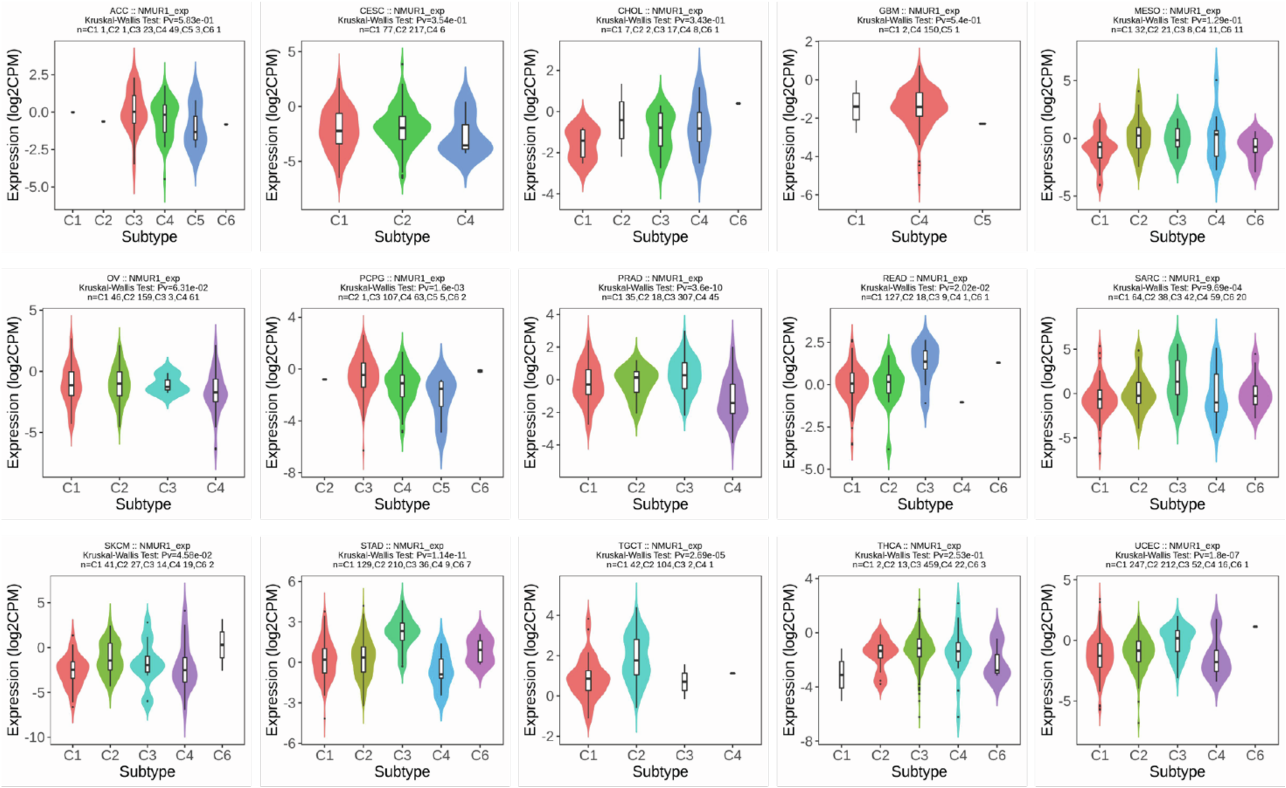

Figure S3. NMUR1 expression levels across different immune subtypes in pan-cancer, Related to Figure 3. Analysis of NMUR1 expression associations with immune subtypes in various cancers, as sourced from the TISIDB database. The p value was calculated using Kruskal-Wallis test.

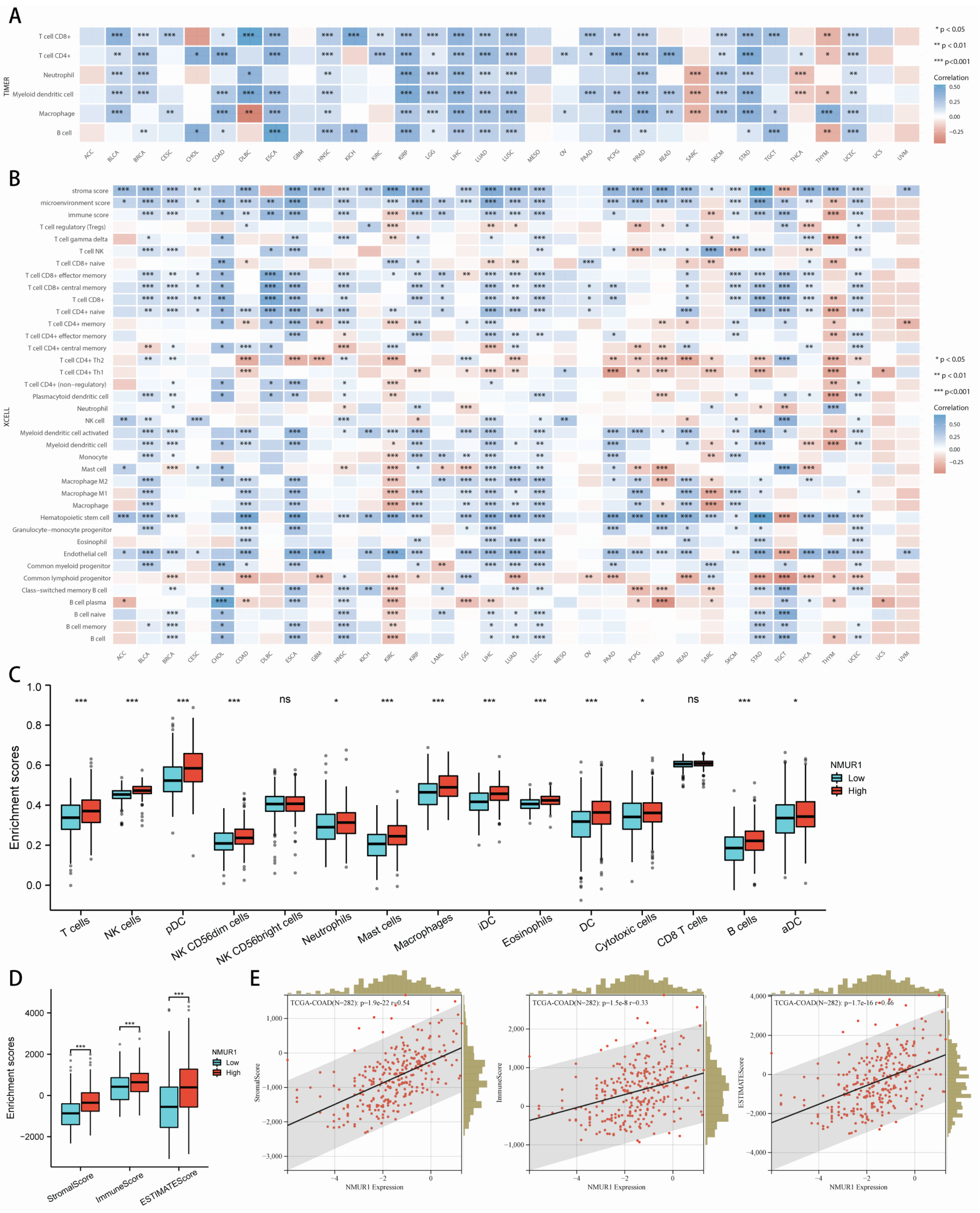

Figure S4. Correlation of NMUR1 expression with immune cell infiltration across cancers, Related to Figure 3. (A) Analysis of the correlation between NMUR1 expression and six immune cell types using the TIMER database. The p value was calculated using Spearman correlation test. (B) Correlation assessment of NMUR1 expression with immune cell

infiltration using the xCELL database. The p value was calculated using Spearman correlation test. (C) Comparison of immune cell infiltration enrichment scores between high and low NMUR1 expression groups in the TCGA-COAD dataset. The p value was calculated using Wilcoxon test. (D) Differential stromal, immune, and ESTIMATE scores in high versus low NMUR1 expression groups within the TCGA-COAD immune cell context. The p value was calculated using Wilcoxon test. (E) Positive correlations between NMUR1 expression levels and stromal, immune, and ESTIMATE scores in the TCGA-COAD dataset. The p value was calculated using Spearman correlation test. ns, no significant; *$p < 0.05$; **, $p < 0.01$; ***, $p < 0.001$.

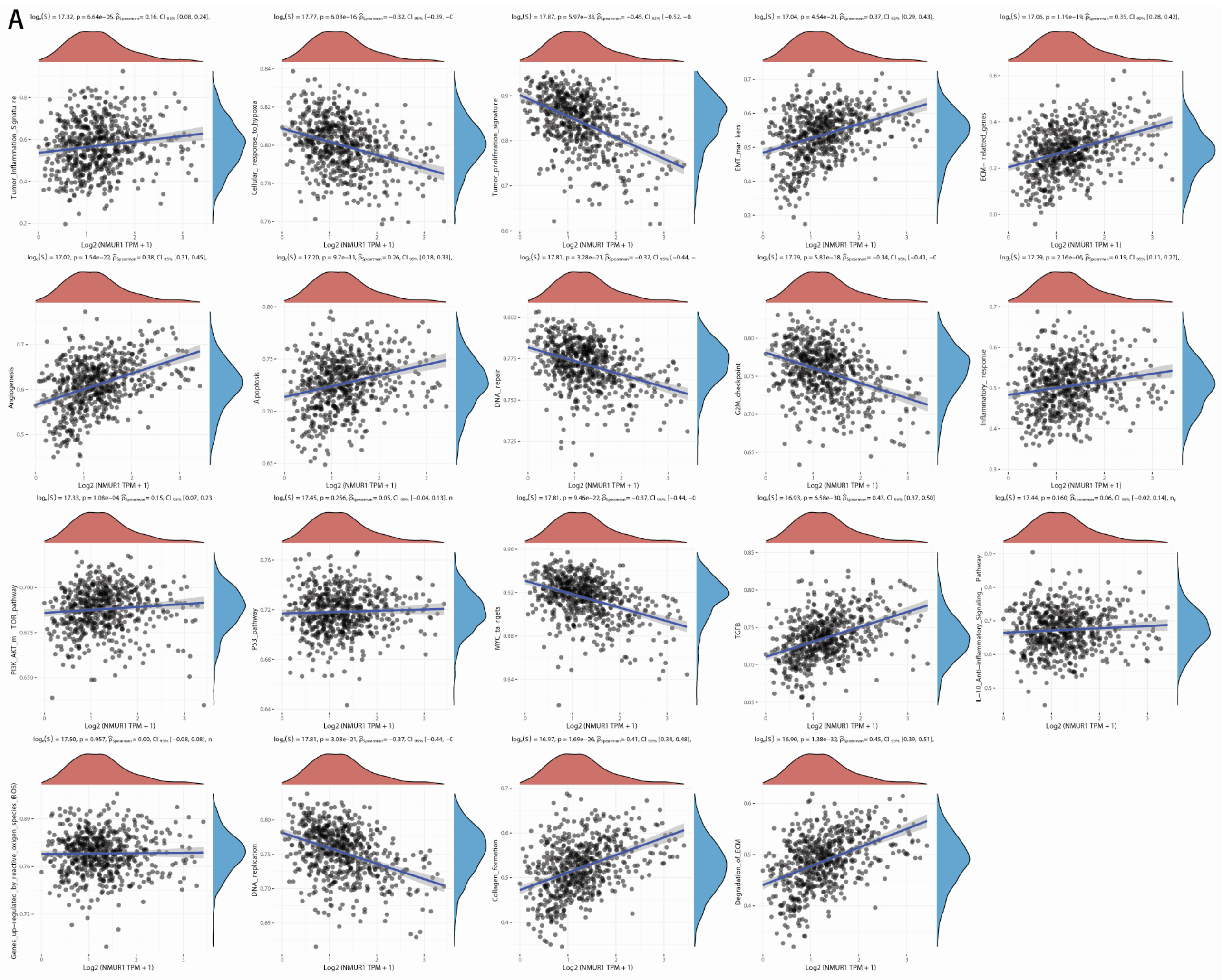

Figure S5. Association of NMUR1 expression with pathway enrichment, Related to Figure 3. (A) Utilizing the TCGA-COAD dataset, we performed Spearman correlation analyses to evaluate the relationship between NMUR1 expression and enrichment scores of 19 cancer-related pathways. The p value was calculated using Spearman correlation test.

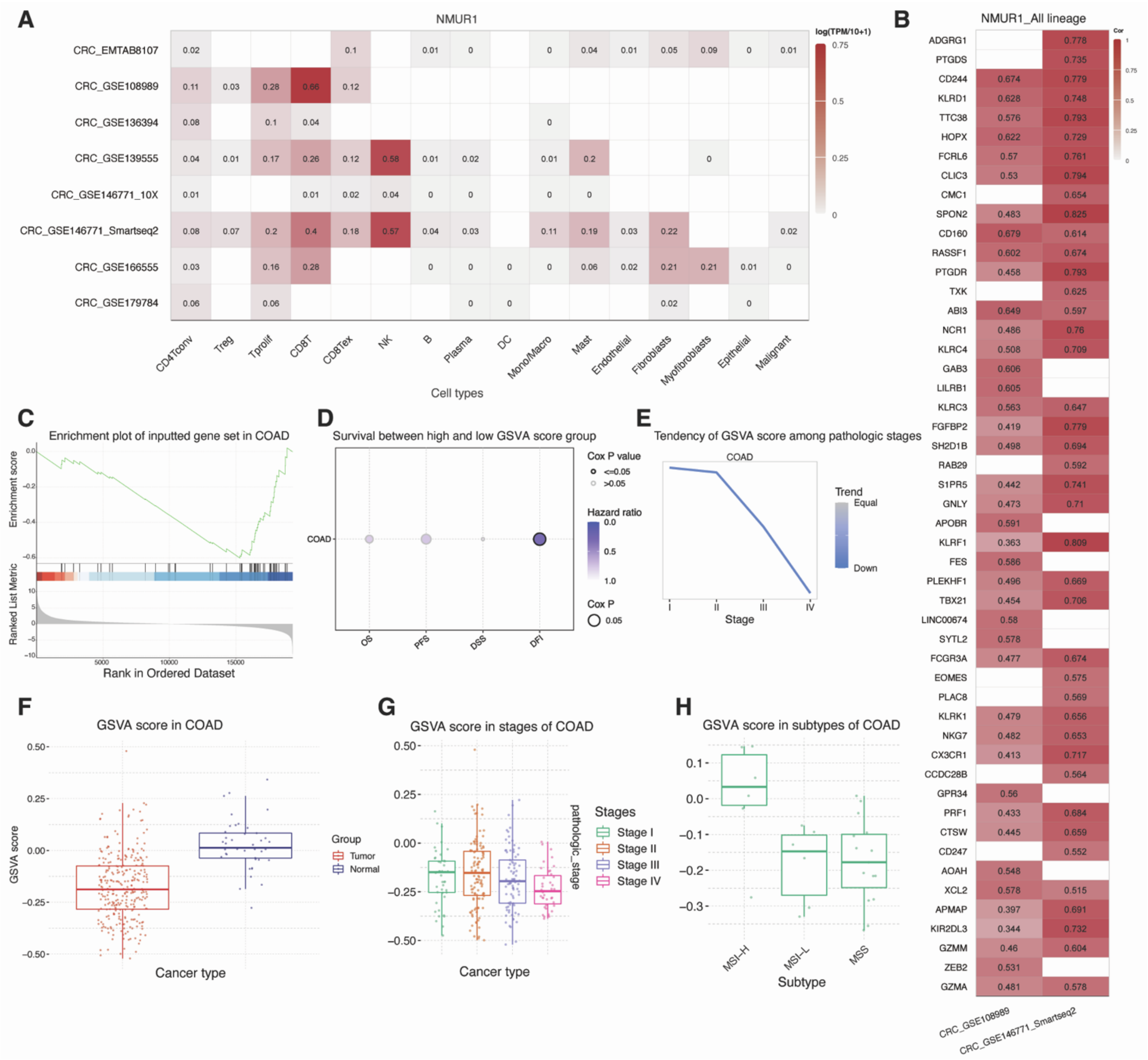

Figure S6. NMUR1 correlated gene sets in CRC as revealed by single cell analysis, Related to Figure 4. (A) Heatmap showcasing the general correlation of NMUR1 expression, with higher levels predominantly in CD8+ T cells and NK cells. (B) The top 50 NMUR1-correlated genes identified from two CRC single-cell datasets. (C) Gene set enrichment plot for NMUR1-correlated genes in COAD. (D) Survival analysis comparing groups with high versus low gene set variation analysis (GSVA) scores. (E) Variation in GSVA scores across different pathological stages in COAD. (F) Comparison of GSVA scores between TCGA-COAD and normal tissue samples. (G) Distribution of GSVA scores across various COAD stages. (H) GSVA score analysis among different COAD subtypes.

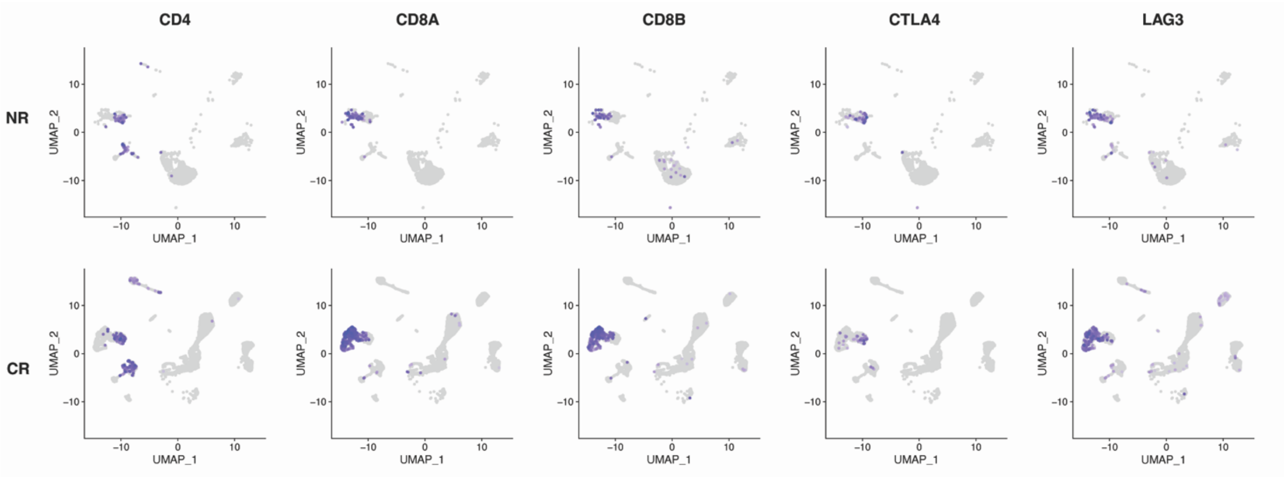

Figure S7. Expression patterns of immune markers in single cell data from CRC patients, Related to Figure 5. Differential expression of immune markers CD4, CD8A, CD8B, CTLA4, and LAG3 in non-responders (NR) versus complete responders (CR) visualized in UMAP plots.

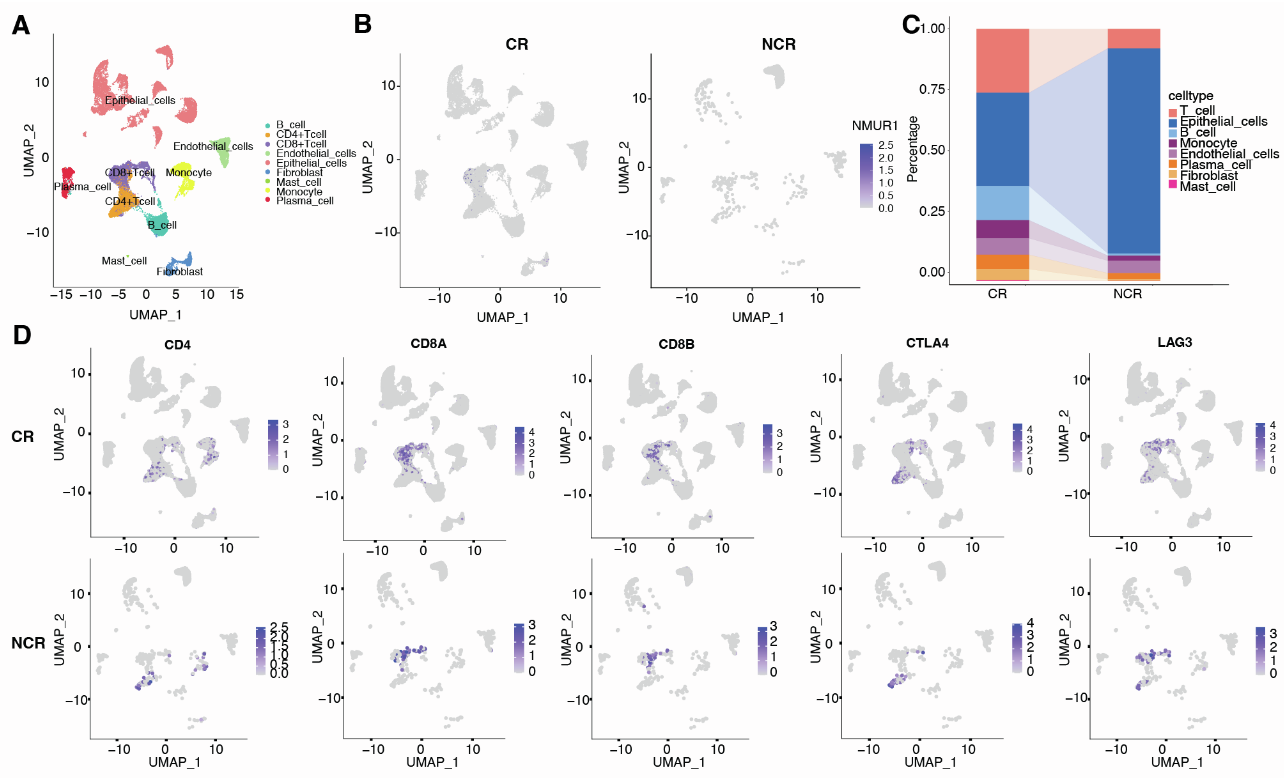

Figure S8. Assessing NMUR1 as an immunotherapy response biomarker in GSE205506, Related to Figure 5. (A) Cell populations annotated in UMAP visualizations of single-cell data from 10 CRC patients. (B) NMUR1 expression comparison between non-complete responders (NCR) and complete responders (CR). (C) Proportional differences in cell types between NCR and CR groups. (D) UMAP visualization of CD4, CD8A, CD8B, CTLA4, and LAG3 expression in NCR and CR patients.

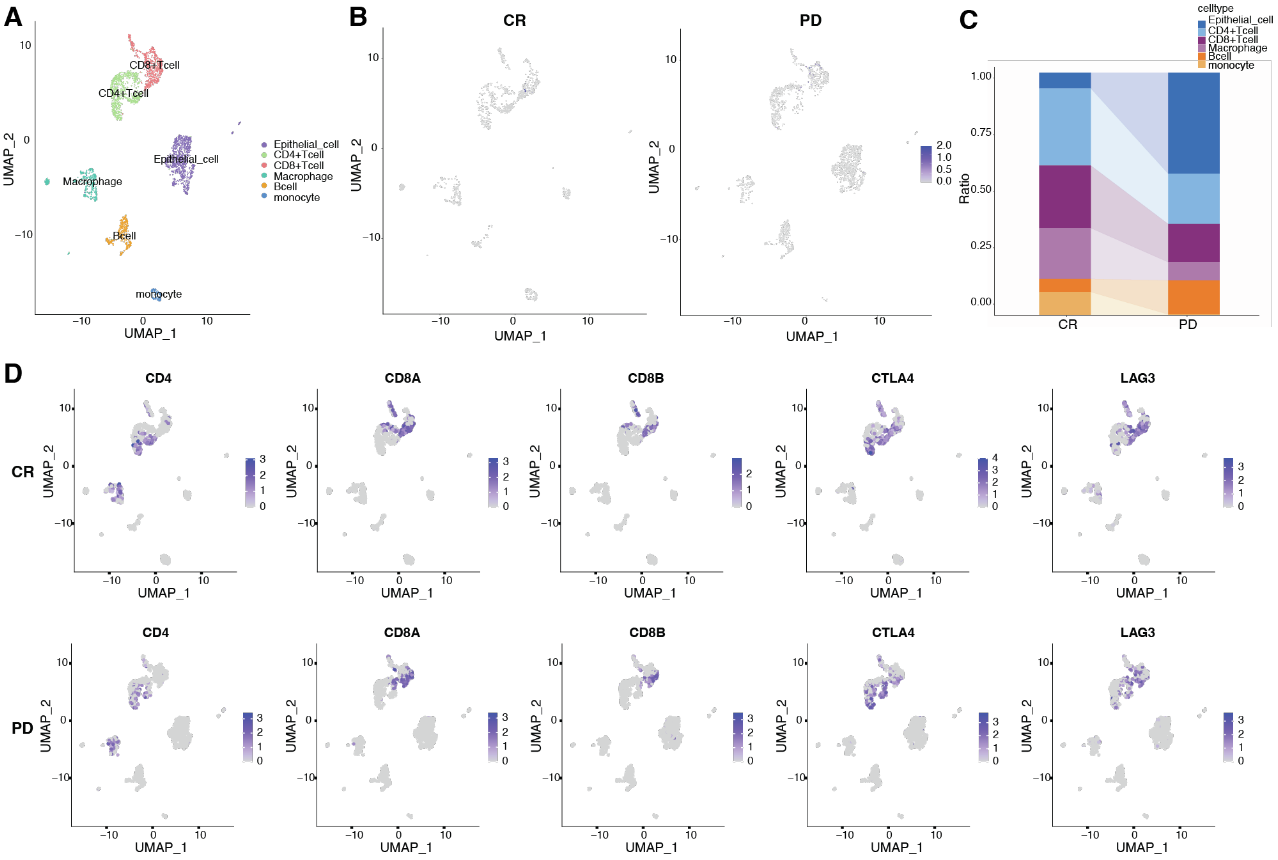

Figure S9. Assessing NMUR1 as an immunotherapy response biomarker in GSE222300, Related to Figure 5. (A) Cell populations annotated in UMAP visualizations of single-cell data from 2 CRC patients. (B) NMUR1 expression comparison between progressive disease (PD) and complete responders (CR). (C) Proportional differences in cell types between PD and CR groups. (D) UMAP visualization of CD4, CD8A, CD8B, CTLA4, and LAG3 expression in PD and CR patients.

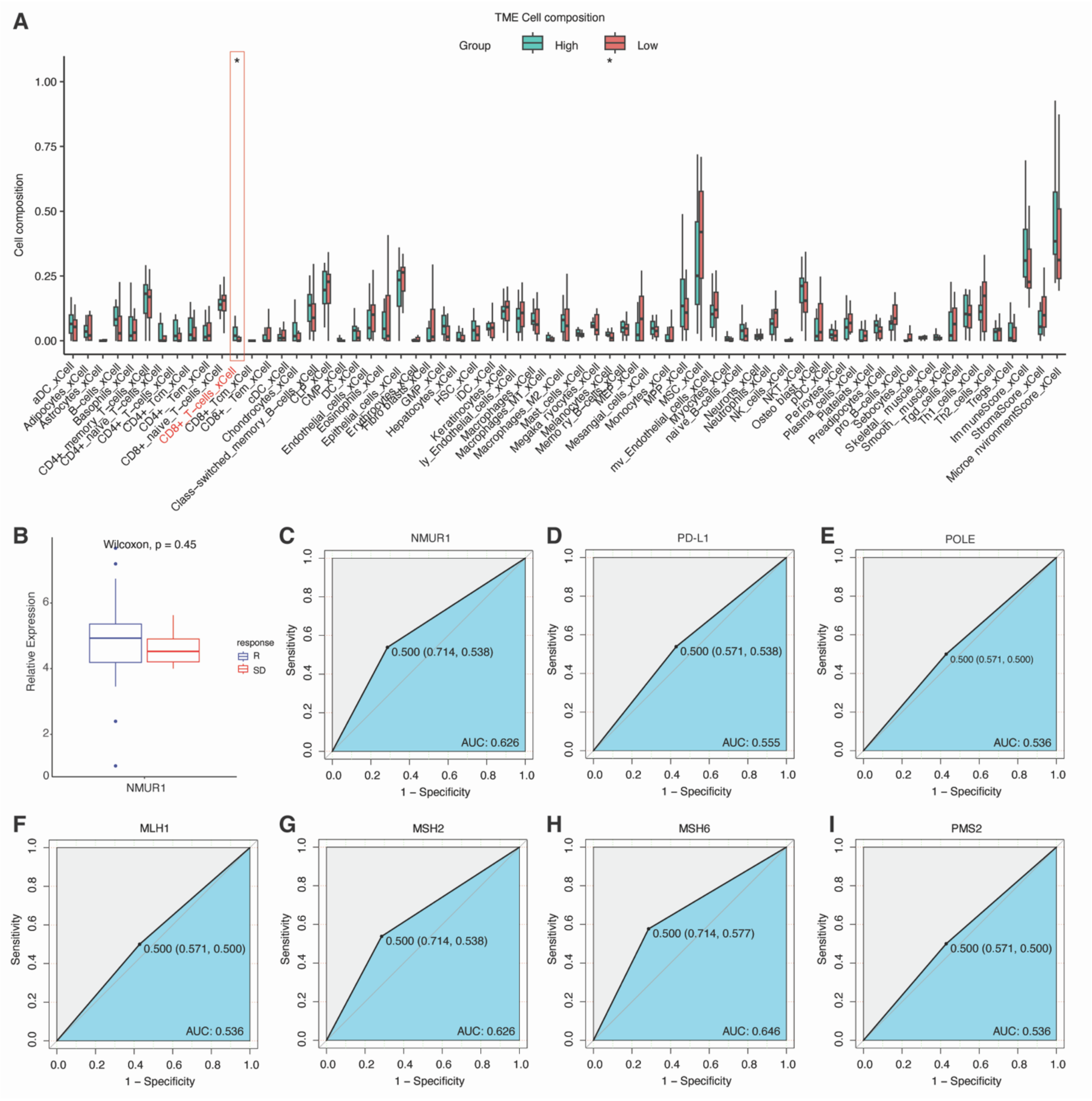

Figure S10. Assessing NMUR1 as an immunotherapy response biomarker in GSE235919, Related to Figure 6. (A) Correlation between NMUR1 and immune cells infiltration by xCELL algorithm. The p value was calculated using Wilcoxon test. (B) Comparison of NMUR1 expression between patients who responded to immunotherapy (R) and those with stable disease (SD). The p value was calculated using Wilcoxon test. (C-I) AUC plots comparing NMUR1 and other potential biomarkers.
